# Gradients of bacteria in the oceanic water column reveal finely-resolved vertical distributions

**DOI:** 10.1371/journal.pone.0298139

**Published:** 2024-04-02

**Authors:** Rachel C. Harbeitner, Fabian Wittmers, Charmaine C. M. Yung, Charlotte A. Eckmann, Elisabeth Hehenberger, Marguerite Blum, David M. Needham, Alexandra Z. Worden

**Affiliations:** 1 Department of Ocean Sciences, University of California Santa Cruz, Santa Cruz, CA, United States of America; 2 Ocean EcoSystems Biology Unit, RD3, GEOMAR Helmholtz Centre for Ocean Research Kiel, Kiel, DE, Germany; 3 Marine Biological Laboratory, Woods Hole, MA, United States of America; 4 Monterey Bay Aquarium Research Institute, Moss Landing, CA, United States of America; Stazione Zoologica Anton Dohrn, ITALY

## Abstract

Bacterial communities directly influence ecological processes in the ocean, and depth has a major influence due to the changeover in primary energy sources between the sunlit photic zone and dark ocean. Here, we examine the abundance and diversity of bacteria in Monterey Bay depth profiles collected from the surface to just above the sediments (e.g., 2000 m). Bacterial abundance in these Pacific Ocean samples decreased by >1 order of magnitude, from 1.22 ±0.69 ×10^6^ cells ml^-1^ in the variable photic zone to 1.44 ± 0.25 ×10^5^ and 6.71 ± 1.23 ×10^4^ cells ml^-1^ in the mesopelagic and bathypelagic, respectively. V1-V2 16S rRNA gene profiling showed diversity increased sharply between the photic and mesopelagic zones. Weighted Gene Correlation Network Analysis clustered co-occurring bacterial amplicon sequence variants (ASVs) into seven subnetwork modules, of which five strongly correlated with depth-related factors. Within surface-associated modules there was a clear distinction between a ‘copiotrophic’ module, correlating with chlorophyll and dominated by e.g., Flavobacteriales and Rhodobacteraceae, and an ‘oligotrophic’ module dominated by diverse Oceanospirillales (such as uncultured JL-ETNP-Y6, SAR86) and Pelagibacterales. Phylogenetic reconstructions of Pelagibacterales and SAR324 using full-length 16S rRNA gene data revealed several additional subclades, expanding known microdiversity within these abundant lineages, including new Pelagibacterales subclades Ia.B, Id, and IIc, which comprised 4–10% of amplicons depending on the subclade and depth zone. SAR324 and Oceanospirillales dominated in the mesopelagic, with SAR324 clade II exhibiting its highest relative abundances (17±4%) in the lower mesopelagic (300–750 m). The two newly-identified SAR324 clades showed highest relative abundances in the photic zone (clade III), while clade IV was extremely low in relative abundance, but present across dark ocean depths. Hierarchical clustering placed microbial communities from 900 m samples with those from the bathypelagic, where Marinimicrobia was distinctively relatively abundant. The patterns resolved herein, through high resolution and statistical replication, establish baselines for marine bacterial abundance and taxonomic distributions across the Monterey Bay water column, against which future change can be assessed.

## Introduction

Patterns in marine bacterial community structure are observable spatially (e.g., latitude, longitude, depth) and temporally [1–4]. Depth and co-associated parameters are major factors in microbial community variations occurring between the surface and deep ocean [5, 6]. A multi-depth view provides the opportunity to identify differences in community structure, potentially highlighting the diversity associated with distinct ecological niches influenced by the environmental conditions at each depth.

Pelagibacterales (commonly referred to as SAR11) is an alphaproteobacterial lineage for which extensive characterization is available. Pelagibacterales cells are heterotrophic and have limited metabolic flexibility because of genome streamlining and subsequent loss of adaptive genes [7, 8], requiring pyruvates, amino acids (or their respective precursors) as well as hydroxymethyl-2-methylpyrimidine for growth [9, 10]. Pelagibacterales are highly abundant in the oceanic water column, comprising on average about one-third of bacterial cells and up to 50% at times in surface waters. Their presence extends to depths below the photic zone, where they can make up to 25% of bacterial cells [11, 12]. Distinct ecotypes or clades exhibit specific annual cycles in depth-related distributions that correlate with seasonal mixing, blooms, and stratification in the photic versus upper-mesopelagic, defined in that study as 0–120 m and 160–300 m, respectively [13–15]. One particular Pelagibacterales clade, referred to as SAR11 clade II, is considered abundant and diverse on sinking particles at deeper depth, while other SAR11 clades and subclades exhibit differences in relative abundance over geographical regions and seasons [13, 16–19]. Environmentally-mediated selection is considered crucial in shaping the biogeography of SAR11 subclades [20].

Like Pelagibacterales, SAR324 [21]—formally also known as Marine Group B [22] and part of Deltaproteobacteria, but now considered their own phylum [23]—are considered ubiquitous in the ocean, and particularly abundant below the photic zone [24–26]. This uncultivated group likely represents organisms with varied genomic content, corresponding potentially to considerable metabolic flexibility, and ecological niches. Some SAR324 genomes contain ribulose-1, 5-bisphosphate carboxylase-oxygenase and sulfur oxidation genes, suggesting the potential for autotrophic CO_2_ fixation coupled with reduced sulfur compound oxidation [27–29]. SAR324 also appears capable of photoheterotrophy and alkane oxidation especially in surface layers, capabilities that potentially underpin the ubiquity of SAR324 as a whole [29, 30]. Understanding the distributions of different groups within SAR324, paired with their apparent metabolic capacity, will allow identification and hypotheses on the ecological niches occupied by SAR324 taxa.

Here, we examine the distribution of bacterial taxa and transitions in diversity throughout the Monterey Bay water column in the eastern North Pacific. Specifically, we sampled depths from 2 to 1,978 m during three expeditions conducted over two seasons, during two years, and investigated bacterial communities using V1-V2 16S rRNA gene amplicon analyses and flow cytometry. Our molecular analyses of the bacterial community used a two-part approach combining a Weighted Gene Correlation Network Analysis (WGCNA) of ASVs with phylogenetic analyses of two of the primary bacterial groups observed (Pelagibacterales and SAR324). These analyses advance our understanding of how different clades within bacterial groups are distributed throughout the water column and reveal variations in marine microbial community composition over space and time in a Pacific marine canyon ecosystem.

## Material & methods

### Oceanographic sampling

**Fig 1:**
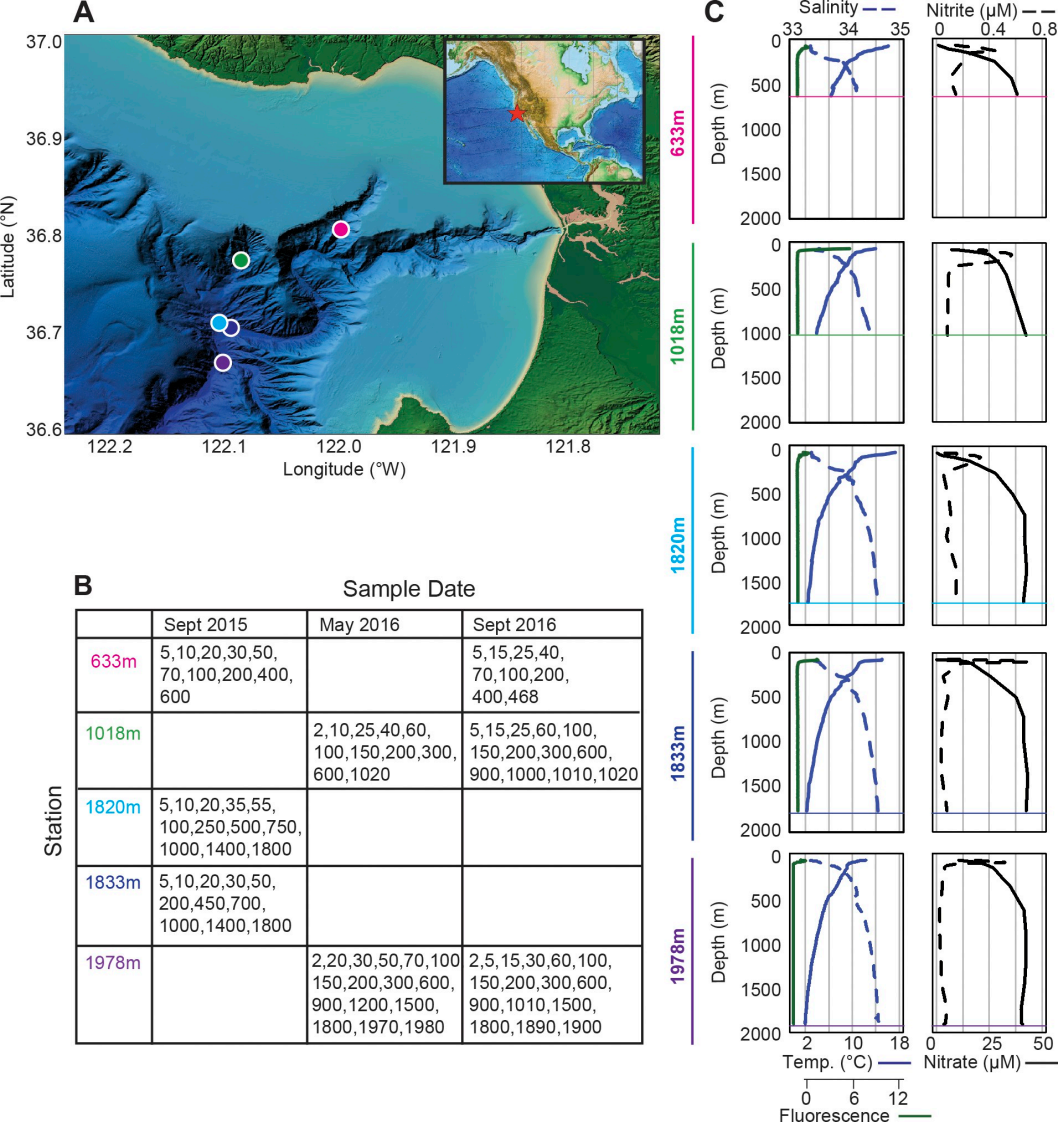
Sampling locations and environmental characteristics throughout the water column. (A) Seafloor bathymetry is indicated in the region of sampling (Monterey Bay) with stations indicated (circles). The inset shows the location of the sampling region within a broader context of the eastern North Pacific. (B) Table of sampling periods for the individual stations (see S1 Table for latitudes, longitude, and other metadata). The depths listed indicate those for which sequencing was performed (16S V1-V2 rRNA gene amplicons). (C) Temperature, salinity, and *in vivo* chlorophyll fluorescence (left panels) and nitrate and nitrite (right panels) representative profiles from each site. Data for these and additional profiles and parameters are provided in the supplementary tables.

R/V *Western Flyer* cruises were in September 2015, May 2016, and September 2016 (Fig 1A, 1B and S1 Table). Five stations were sampled: 1978m, 1833m, 1820m, 1018m, and 633m, for which the numbers reflect the station bottom depth. Seawater was collected at 12 to 16 depths from the surface to near the seafloor using Niskin bottles mounted on a conductivity-temperature-depth rosette or the remotely-operated vehicle (ROV) *Doc Ricketts*. Collections were distributed across classically defined depth zones, i.e., the photic (40% of all samples), mesopelagic (34%), and bathypelagic (26%) zones (S1 Table). DNA samples were collected by filtering 1 L of seawater onto a 0.2 μm 47 mm Supor filter (Pall Gelman, East Hills, NY, USA) with immediate freezing at -80°C. Flow cytometry samples were preserved [31] and nutrients were sampled and analyzed as previously described [32].

### Flow cytometry

Bacterial cell counts were measured by flow cytometry using an Influx (BD Biosciences, San Jose, CA, USA) with a 200 mW 488 nm laser. Thawed samples were stained with SYBR Green I (0.5X final concentration; Molecular Probes, Inc., Eugene, OR, USA) for 15 min at room temperature in the dark [33]. 0.75 μm yellow-green and 0.5 μm green polystyrene beads (Polysciences, Inc., Warrington, PA, USA) were added as a standard. Samples were run for 2 min at 25 μl min^-1^ after a 2 min pre-run, and the inline measurement was confirmed by weighing the sample before and after the run. Bacterial populations were resolved by green fluorescence (520/35 nm bandpass filter, also serving as the data collection trigger) and Forward Angle Light Scatter (FALS). Cyanobacteria were analyzed in unstained samples based on FALS (trigger) versus chlorophyll autofluorescence (692/40 nm bandpass) and phycoerythrin autofluorescence (572/27 nm bandpass, *Synechococcus* only). *Prochlorococcus* abundances from unstained samples were subtracted from bacterial abundances derived from stained samples to enumerate non-photosynthetic bacteria. Flow cytometry data were analyzed in WinList 3D 8.0 (Verity Software House, Topsham, ME, USA).

### DNA extraction, PCR and sequencing

DNA was extracted from filters as described previously [34], and quantified with the QuBit dsDNA high-sensitivity assay (Invitrogen, Carlsbad, CA, USA). Prior to PCR, templates were diluted to 5 ng μl^-1^ with TE pH 8 (Thermo Fisher Scientific, Waltham, MA, USA). PCRs (50 μl) were set up [35] and cycled [31] using the primers 27FB, 5′-AGRGTTYGATYMTGGCTCAG-3′ and 338RPL, 5′- GCWGCCWCCCGTAGGWGT-3′ [36]. Following PCR, an aliquot was run on a 1% agarose gel to verify the desired V1-V2 16S rRNA gene product (∼300 nt) had been amplified. Sequencing using Illumina MiSeq (2x300 bp paired-end reads) was performed at the University of Arizona Genetics Core.

### V1-V2 16S rRNA gene amplicon processing

Low-quality bases were trimmed from the sequences using a 10% read length window with a Q25 running-quality threshold with sickle (v1.33) [37]. Amplicons were processed with USEARCH [38]. Paired-end sequences were merged with a ≥50-nucleotide overlap, minimum sequence length of 200 nucleotides, and maximum 5% mismatches. Merged sequences were identified as being of low quality when having *E*_*max*_ >1 and subsequently discarded per recommendations of the tool developer. Primers were removed with cutadapt [39]. For each sample, between 38,824 and 460,250 amplicons were generated that passed quality control measures (median = 178,675, n = 97; S2 Table).

Merged amplicon sequences were resolved into ASVs of 100% pairwise nucleotide identity with unoise3 [40] as implemented in USEARCH (v10.0.240) [38]. Taxonomy was assigned using the SILVA database (release 128) [41] via the QIIME2-2018.2 q2-feature-classifier [42] and mitochondrial, plastid, and cyanobacterial ASVs excluded, resulting in 57,827 total bacterial ASVs. The V1-V2 16S rRNA primers used do not recover Archaea [43]. Raw reads were deposited in NCBI-Short Read Archive under the Bioproject number PRJNA643269.

### Statistical and diversity analyses

Bacterial community ASV analyses were conducted using the “vegan” package (v2.4.4) in R (v3.4.1) [44]. Rarefaction curves and final slopes were computed using the vegan rareslope function. For each sample, the Shannon diversity index was calculated to estimate community diversity, this index was calculated on both rarefied and non-rarefied data, resulting in the same trends. ASV relative abundances were calculated by dividing the ASV count by the total number of bacterial amplicons and log2 transformed for visualization. Community composition (rarefied data) based on ASV relative abundances was compared between samples using Bray-Curtis dissimilarity and visualized using hierarchical clustering. Uncertainty of the hierarchical cluster analysis was analyzed using the R package “pvclust” (v.2.2–0), implementing multiscale bootstrap resampling to provide an approximately unbiased p-value and bootstrap probability value for each cluster [45]. Plots were generated using R package “ggplot2” (v2.2.1) [46]. Weighted Gene Correlation Network Analysis (WGCNA) was performed using the R package “WGCNA” (v1.70–3) [47]. WGCNA was used to find clusters (modules) of highly correlated taxa across samples and relate these clusters to each other and environmental parameters. Additionally, a non-metric multidimensional scaling (NMDS) plot using Bray-Curtis dissimilarity was constructed in vegan using the rarefied samples for which complete environmental data was available (85 samples).

### Pelagibacterales and SAR324 phylogenetic reconstructions and amplicon placement

A total of 137 near full-length 16S rRNA gene sequences representing Pelagibacterales were retrieved from the SILVA database (release 128) and through blastn (v2.6.0) [48] searches of GenBank. These sequences and one *Escherichia coli* and one *Magnetococcus marinus* (as distant outgroup members) were aligned using MAFFT (v7.402) [49] with default parameters, and ambiguously aligned sites were removed using trimAl (v1.4) with no gaps allowed [50]. Phylogenetic inferences were performed using Maximum Likelihood methods implemented in RAxML (v8.2.10) [51] under the gamma-corrected GTR model of evolution based on 1,303 positions, followed by 1,000 bootstrap replicates. The final tree was visualized with FigTree (v1.4.0).

For SAR324 phylogenetic analyses, 88 near full-length 16S rRNA gene sequences were retrieved by GenBank blastn [48] searches. These alongside three Nitrospinaceae, two Desulfobulbaceae, and three Desulfovibrionaceae (as an outgroup) were aligned with MAFFT [49] using the L-INS-I algorithm and ambiguously aligned sites were removed using trimAl with a gap threshold of 0.3 [50]. Phylogenetic inferences were performed using Maximum Likelihood methods implemented in RAxML [51] under gamma-corrected GTR model of evolution based on 1,227 positions, followed by 1,000 bootstrap replicates. The final tree was visualized with FigTree (v1.4.0).

Amplicons were phylogenetically placed using PhyloAssigner [17] by first retrieving Pelagibacterales amplicons based on placement on a previously developed global 16S rRNA gene reference tree [17]. This set was then run in PhyloAssigner using the above Pelagibacterales reference tree to parse amplicons into identified clades and subclades. SAR324 amplicons were also retrieved based on their taxonomic assignment in the global 16S rRNA gene reference tree [17], and placed using PhyloAssigner and our SAR324 reference tree to assign amplicons to SAR324 clades. To further examine Pelagibacterales distributions, relative abundance of sequences placed in each subclade was calculated and hierarchical clustering performed based on Bray-Curtis similarity of Pelagibacterales relative abundances per sample to compare community composition across samples. The same analyses were performed for SAR324 amplicons. Relative abundances were log2 transformed for visualization and plots were generated using R package “ggplot2” [46].

## Results and discussion

### Oceanographic conditions

Expeditions were performed in Monterey Bay, located in the eastern North Pacific and encompassing a submarine canyon system with the deepest sites in the outer bay and shallowest in the inner canyon zone (Fig 1). Profiles from stations sampled more than once were similar between years, apart from the photic zone (S1 Table, S1 Fig). The latter is known to be highly dynamic, with varying influences of both the eastern boundary current and upwelling extent [52, 53]. During our study, the photic zone ranged from between 25 m (Stn. 1978m; May 2016) and 150 m (Stn. 1820m; Sept 2015), and subsurface chlorophyll maxima were generally not observed, indicating well-mixed surface waters (Fig 1C, S1 Table). High variability was also reflected in chlorophyll-*a* concentrations, which ranged from a minimum 0.84 mg m^-3^ to maximum 13.78 mg m^-3^ in surface waters between years (S1 Table), similar to prior studies [32, 52].

Water temperatures ranged from 12.13 to 17.26°C at the surface, and from 2.05°C (Stn. 1978m) to 6.09°C (600 m; Stn. 633m) near the seafloor (Fig 1C, S1 Table). Stations with bottom depths >1500 m were consistently colder near the seafloor (2.05–2.38°C) than the others (3.72–6.09°C). Salinity ranged from 33.3 to 34.6, increasing with depth, while oxygen decreased (S1 Table). Phosphate, silicate, and nitrate concentrations generally increased with depth but exhibited different gradient patterns. Photic zone phosphate concentrations ranged from 0.36 to 2.45 μM; however, regardless of station bottom depth, concentrations near the seafloor ranged from 2.65 to 3.63 μM across all samplings and were generally between 2.27 and 3.94 μM in the dark ocean (S1 Table). Silicate increased by over two orders of magnitude between the surface and the deepest sites, with the steepest increase in the upper mesopelagic ranges (S1 Table). A subsurface nitrite maximum was observed that shifted in depth, with the shallowest being 15 m (Stn. 1018m; Sept 2016) and deepest 70 m (Stn. 633m; Sept 2015) (Fig 1C and S1 Table).

Together these oceanographic conditions indicate that the study area is broadly representative of relatively deep waters of the eastern North Pacific and its adjacent upwelling areas, and indeed of eastern boundary upwelling ecosystems in general [54]. Sampling and analysis of the entire water column, and similar profiles (especially in the deep samples), across two time-points sets the stage for understanding how these conditions impact microbial communities. These collections capture communities associated with relatively variable productivity at the surface, apparently more stable, deep waters, and variable transitional depths in between. Specifically, the state of relatively high phytoplankton productivity and production of labile organic carbon in the surface layers fuels rapid growth of heterotrophic bacteria. As this material sinks through the water column, organic carbon is consumed, while in parallel temperature and light decrease (light decreasing to zero below the photic zone). At the same time inorganic nutrients increase due to remineralization processes, and at the deepest depths bacteria with metabolisms suited for cold, low and/or recalcitrant organic carbon flourish [55]. To date, the degree to which these communities vary between broadly similar profiles in such an eastern boundary current system is poorly characterized, especially relative to systems such as the sub-tropical Atlantic and tropical central Pacific [56].

### Bacterial cell abundance

**Fig 2:**
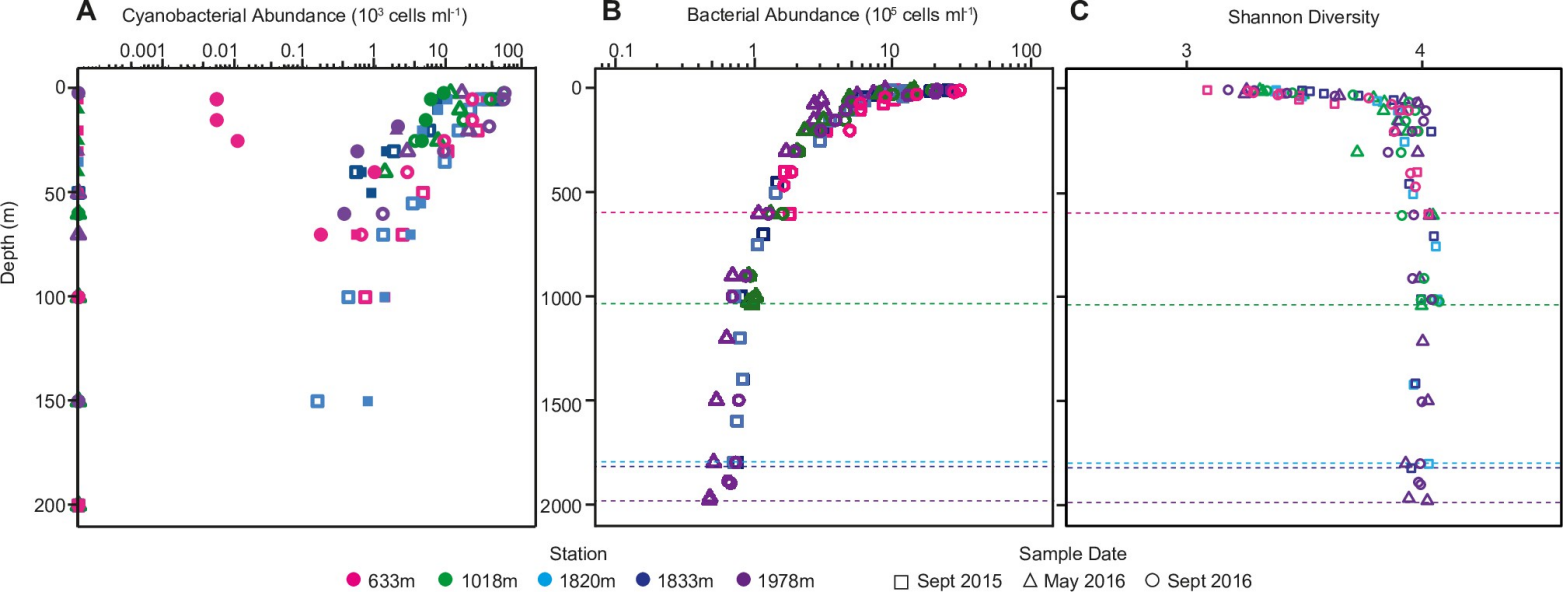
Abundance and diversity of bacteria and cyanobacteria across depth and sample periods. (A) Cell abundance of *Prochlorococcus* (closed symbols) and *Synechococcus* (open symbols) in the upper 200 m as enumerated by flow cytometry; cyanobacterial cells were not detected below this depth. (B) Heterotrophic bacterial cell abundance (i.e., non-pigmented; analyzed by flow cytometry after SYBR Green I staining) decreased with depth by 1.5 orders of magnitude. Replicates from individual sampling dates revealed a variance of 16% (n = 8) that can be attributed to methodology. (C) Shannon diversity indices for all sequenced samples and depth profiles as calculated from V1-V2 16S rRNA gene ASV data. September 2015 (squares), May 2016 (triangles), and September 2016 (circles) sampling dates are indicated, while stations are represented by color for all panels. Horizontal dotted lines (plots B and C) represent the bottom depth for the respective stations.

To establish bacterial abundances, both cyanobacteria (*Prochlorococcus* and *Synechococcus*) and heterotrophic bacteria (i.e., non-pigmented) were enumerated using either natural autofluorescence characteristics or DNA staining, respectively, as well as scatter properties as ascertained by flow cytometry (Fig 2A, 2B and S1 Table). The abundance of heterotrophic bacteria varied 10-fold in the photic zone between stations and periods sampled (Fig 2B and S1 Table), such that their cell abundances ranged from 3.06×10^5^ cells ml^-1^ (50 m; Stn. 1978m; May 2016) to 3.05×10^6^ cells ml^-1^ (5 m; Stn. 633m; September 2016). Between the photic zone (on average 1.22×10^6^±6.92×10^5^ cells ml^-1^) and the deepest depths analyzed (on average 6.71×10^4^±1.23×10^4^ cells ml^-1^), heterotrophic bacterial abundances decreased by 1.5 orders of magnitude. Closest to shore (Stns. 633m and 1018m; Fig 1A and S1 Table), we observed significantly higher cell abundances overlying the seafloor (1.35×10^5^±4.32×10^4^ cells ml^-1^) compared to offshore stations with deeper bottom depths (6.51×10^4^±1.23×10^4^ cells ml^-1^; *t*-test: *P*<0.001). Abundance also increased rapidly moving upwards through dark waters approaching the photic zone at Stns. 633m and 1018m (S1 Table). These vertical trends are similar to those reported at the San Pedro Time-series, SPOT, which is also located in the eastern North Pacific (~480 km from Monterey Bay) and has a bottom depth of ~900 m [3]. The rate of reduction in cell concentration was slower at depth than in shallower samples at our deeper stations (Fig 2B). Reduced abundances of heterotrophic bacteria at deeper depths have been established in regional studies [55] and more global circumnavigations [2] as well as meta-analyses [55, 57]. This decrease in cellular abundance reflects differences in organic material available for growth, which is more abundant (and more labile) in the surface due to the activities of phytoplankton; differences in heterotrophic bacterial abundances also correspond to decreases in bacterial productivity with depth [55]. Here, cyanobacteria were present only above 150 m and *Synechococcus* was maximally 7.24×10^4^ cells ml^-1^ in the photic zone (2 m; Stn. 1978m; September 2016), while the *Prochlorococcus* maximum cell abundance was 1.08×10^4^ cells ml^-1^ (5 m; Stn. 1820m; September 2015; Fig 2A and S1 Table). Further, *Prochlorococcus* was not always detected, and is generally found to be low in abundance or undetectable in the inner bay, whereas *Synechococcus* appears to be omnipresent and more abundant [31, 58, 59]. A t-test revealed a weak correlation of heterotrophic bacterial cell abundances with *Prochlorococcus* (0.47, *P*<0.0001) and a stronger correlation with *Synechococcus* (0.82, *P*<0.0001).

### Bacterial diversity

To examine bacterial diversity throughout the water column, we sequenced and analyzed an average of 183,099±76,142 16S rRNA gene amplicons per sample, which resulted in a total of 57,827 ASVs including singletons (44,260 excluding singletons). The V1-V2 16S rRNA variable region used has previously been implemented to resolve taxonomic groups of marine bacteria [36, 43], including diversity within Pelagibacterales [17]. First, we performed rarefaction analyses on the amplicons and ASVs generated for each sample for which computation of the slope on a sliding window indicated saturation for all samples (final slope- mean: 0.00918; median: 0.00819), based on Ocean Sampling Day (OSD) cutoffs [60]. Samples from Stn.1978m at 1800 m depth and Stn. 1018m at 300 m depth, although outliers with final slopes of only 0.034 and 0.025, were still saturated (S1A and S1B Fig). We also compared the Shannon Diversity index computed for each sample with the number of amplicons per sample and found no correlation between these two factors (S2 Fig). Taking the whole set together, the Shannon Diversity indices showed clear depth-related changes in bacterial diversity levels, with alpha diversity increasing with depth, such that it was consistently higher below the photic zone, and generally increased across photic zone depths below the very surface. The sharpest increase in bacterial diversity occurred near the base of the photic zone and remained relatively consistent across depths to the deepest point of sampling (Fig 2C).

Prior studies of vertical trends typically involve amplicon clustering at the 97% OTU level, different from the 100% ASV level used herein, and larger increments between sampling depths. Nevertheless, similar trends were reported for the photic zone to 450–750 m and 1500 m in the northern Gulf of Mexico (V4 16S rRNA gene amplicons, 97% clustering) [61], and from the photic zone to 500 m and 2000 m in the Mediterranean Sea (V6 16S rRNA gene amplicons, 97% clustering) [62]. In contrast, a study of four depths in the western and eastern tropical North Pacific, and western subarctic North Pacific, found the highest bacterial diversity generally between 100 and 500 m, and lower diversity in the surface (10 m) and 2000 m, when using V3-V4 16S rRNA gene amplicons clustered at 97% [63]. Highest bacterial diversity in the mesopelagic has also been reported in two equatorial Pacific and one North Pacific gyre stations (V4-V6 16S rRNA gene amplicons, 97% clustering) with sampling at multiple depths in each depth zone and similar increments between sampled depths as in our study [5]. In addition to these regional studies, molecular diversity estimates of prokaryotes in more global expeditions, such as Tara Oceans, with sampling to 1000 m, and Malaspina, with a bottom sample at 4000 m, show that diversity increases with depth, although at low vertical resolution in the dark ocean [2, 64, 65].

The observed increase in bacterial diversity below the photic zone implies that the number of niches realized by prokaryotes is larger at depth. The mechanisms behind this are still unclear, but have been proposed to connect to the wide diversity of metabolic roles related to nitrogen, sulfur, and carbon cycling and other processes carried out by bacteria in the deep sea. Some of these metabolic roles may be more prevalent in the deep sea compared to the surface. Additionally, the presence of particles in our sampling may also play a role in the observed patterns of diversity, as particles have been typically reported to contain a higher diversity of bacteria [66–68], and this phenomenon may increase with depth [12]. Overall, the types and shifts in the proportion of labile versus recalcitrant organic material associated with particles is a function of depth, such that the presence of more diverse organic substrates in deeper waters likely results in the greater variety of bacterial taxa.

### Bacterial community composition delineates depth zones

Classical studies have categorized different parts of the water column based on the resolution of sampling possible–and have identified four major zones: the epipelagic, often termed the photic zone, where sunlight is sufficient to support primary production (0–200 m), the mesopelagic (200 to 1000 m), and the bathypelagic (1000 m to 100 m above the seafloor) [69]. The bathypelagic is sometimes further delineated to define the abyssopelagic, depths below 4000 m, that are not addressed herein. Higher resolution studies based on 16S rRNA gene amplicon analyses have introduced more nuance to the classical depth zone ‘cutoffs’. For example, multi-depth vertical profiling (for multiple years) in the subtropical North Atlantic demonstrates that the top boundary of the upper mesopelagic is often in the vicinity of 160 m rather than 200 m, with important seasonal nuances [13]. Here, we focused on repeat sampling, with greater depth resolution than typically reported, and sample collection distributed through depths that would be classically partitioned as photic, mesopelagic, and bathypelagic.

**Fig 3:**
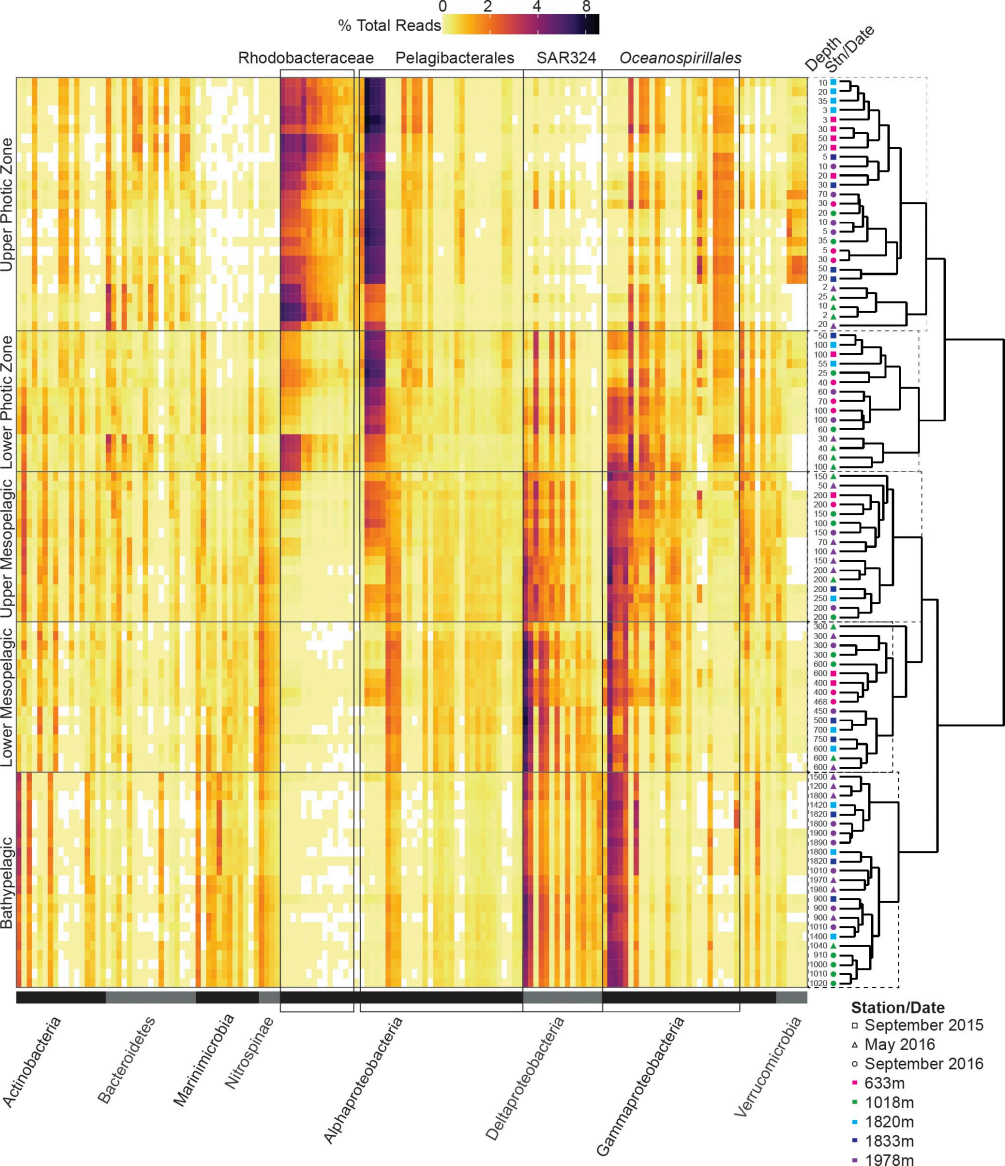
Community and taxonomic patterns in bacterial communities along the depth gradient. The right side shows hierarchical clustering performed on Bray-Curtis dissimilarities of bacterial communities based on the 1000 most relatively abundant ASVs of bacteria (both non-photosynthetic and cyanobacteria (i.e., photosynthetic); see S4 Fig for non-photosynthetic bacteria only). Stations (color) and sampling dates (shape) are indicated as is the depth sampled (m). The left side shows relative abundance of the 50 most relatively abundant ASVs (columns) per sample from the five main depth zones as defined herein. Ordering along the X-axis is by phyla (indicated by horizontal gray bars at the base of figure) and rows represent individual water samples–corresponding to those immediately adjacent (i.e., to the right). ASV relative abundances, sequences, and taxonomic classification are detailed in S3 Table. The four groups with the overall highest relative abundances are indicated (boxes overlaying heat map) and were alphaproteobacterial ASVs assigned to the *Rhodobacteraceae* and *Pelagibacterales* Ia in the photic zone, whereas in mesopelagic and bathypelagic samples ASVs from SAR324 and Gammaproteobacteria *Oceanospirillales* exhibited the highest relative abundances. Taxonomic assignment for ASVs is based on the SILVA database.

We first examined how bacterial community structure connects to the classically predefined zones by clustering samples based on the bacterial community. Specifically, we used the 1,000 most relatively abundant ASVs and Bray-Curtis dissimilarities, to perform hierarchical clustering and statistical testing. Clustering clearly delineated a ‘bathypelagic’ zone that incorporated all samples from 900 m (i.e., shallower than some strict definitions [69] and below (900 to 1980 m, the latter being at our deepest site), and a broad photic zone set (the majority of samples from 100 m and shallower) (Fig 3). Within the latter were two primary clusters that revealed upper (primarily samples from 2 to 30 m) and lower photic zone sets (primarily 35 to 70 m). Adjacent to the nominal bathypelagic cluster were mesopelagic samples (150 to 750 m). These formed two clusters, one containing lower mesopelagic samples (300 to 750), and the other containing upper mesopelagic samples (150 to 250), with the latter also including some samples from shallower depths, likely reflecting the fact that the photic zone in Monterey Bay is highly variable in its depth extent, and at times very shallow [70]. Additionally, in May 2016 there was a distinctive upper photic cluster (2–25 m), with a second similar but still distinct community in the lower photic zone. Site-related or seasonal differences did not stand out within mesopelagic samples, which is in contrast to waters in which the entire water column can be subjected to vertical mixing like in the eastern Mediterranean and subtropical North Atlantic [71, 72]. However, the delineation between upper (again, 150 to 250 m, with occasional shallower samples) and lower (300 to 750 m) mesopelagic were statistically supported, and samples from >750 m depth were excluded from the mesopelagic cluster altogether (S3 Fig). While we do not have the sampling resolution to examine where the shift to bathypelagic communities happens (i.e., between 750 m and 900 m), our findings clearly place 900 m communities, for these Monterey Bay profiles during our time of sampling, with depths ≥1000 m which have been classically defined as the bathypelagic.

With respect to shifts in patterns of bacterial relative abundances, most variability was connected to photic zone variability, as above, and such shifts were not evident in the mesopelagic or bathypelagic over time. For example, increased relative abundances of alphaproteobacterial class Rhodobacteraceae ASVs influenced the composition-based clustering of May 2016 upper photic (2–25 m) samples, making up 24.2±9.3% of this cluster (Fig 3). Additionally, Bacteroidetes show higher relative abundances in the photic zone, with means of 19.0±6.8%, 9.0±3.2%, and 8.4±1.2% in the photic, mesopelagic, and bathypelagic (henceforth depths ≥900 m), respectively (Fig 3). The highest relative abundance of Bacteroidetes ASVs was observed in May 2016 upper photic samples (23.8±5.1%) coincident with the increased relative abundance of Rhodobacteraceae.

Alphaproteobacteria dominated photic amplicons, with Pelagibacterales subclade Ia and Rhodobacteraceae ASVs exhibiting the highest relative amplicon abundances (20.0±6.8% and 16.4±8.2%, respectively) (Fig 3). Contributions from these taxa decreased in the mesopelagic (13.0±2.5% and 2.0±2.7%, respectively) and bathypelagic (7.1±1.1% and 0.4±0.2%, respectively), where SAR324 and the gammaproteobacterial order Oceanospirillales dominated (mesopelagic: 13.6±4.4% and 16.8±4.1%, respectively; bathypelagic: 13.6±2.2% and 16.4±2.5%, respectively). Marinimicrobia (also commonly referred to as SAR406) were mostly present in the bathypelagic (13.6±1.6%), with lower relative abundances in the mesopelagic (8.2±3.3%) and photic (1.8±1.6%) zones. Actinobacteria relative abundances increased slightly through the water column (3.3±1.6% photic, 6.6±1.1% mesopelagic, 7.2±0.9% bathypelagic), and Nitrospinae were lowest in the photic zone (0.9±1.2%) with similar mesopelagic (4.2±0.6%) and bathypelagic (3.2±0.4%) contributions. Verrucomicrobia exhibited similar abundances from the photic (5.0±2.5%), to the mesopelagic (3.3±0.8%) and bathypelagic (4.7±1.0%; Fig 3).

### Strong vertical gradients in abundant bacterial ASVs

We next examined the 50 most abundant ASVs of each of the three major depth zones as identified herein, i.e. photic (0 to 100 m), mesotrophic (150 to 750 m), and bathypelagic (900 to 2000, the latter being the rounded depth of our deepest site (S3 Table; in sum n = 148 ASVs, with some being present in more than one depth zone). ASVs belonging to specific lineages often exhibited depth-related patterns. For example, four of the Oceanospirillales ASVs had higher relative abundances in the mesopelagic (3.7±0.8%, 1.9±0.4%, 1.6±0.6%, 1.3±0.5%) and bathypelagic (3.1±0.3%, 2.8±0.4%, 2.3±0.4%, 1.4±0.3%) than in the photic zone (0.7±0.9%, 0.5±0.6%, 0.7±0.9%, 0.5±0.7%). Just one Oceanospirillales ASV had >1% relative abundance in the photic zone (1.6±0.7%) and exhibited lower relative abundance in the mesopelagic (0.6±0.6%) and bathypelagic (0.02±0.02%) depths. The remaining 19 Oceanospirillales ASVs were present at <0.5% at all depths. Likewise, four distinct Actinobacteria ASVs were observed in the bathypelagic (1.7±0.5%, 0.9±0.3%, 0.7±0.2%, 0.5±0.1%) that exhibited lower relative abundance at photic (0.1±0.1%, 0.1±0.1%, 0.0±0.1%, 0.0±0.0%) and mesopelagic (0.2±0.1%, 0.1±0.1%, 0.1±0.0%, 0.1±0.0%) depths. Three other Actinobacteria from the mesopelagic (0.7±0.5%, 0.6±0.3%, 0.6±0.3%) exhibited lower relative abundance at photic (0.4±0.5%, 0.1±0.1%, 0.1±0.1%) and bathypelagic (0.0±0.0%, 0.1±0.1%, 0.1±0.1%) depths. The two groups highlighted here (Oceanospirillales and Actinobacteria) are widespread in the ocean and have been shown to be abundant throughout the water column [12, 73, 74]. While the functional differences between the sub-species represented by the ASVs assigned to the Oceanospirillales and Actinobacteria are not yet known, these results highlight that even once more genomic information becomes known, there will likely be subtle differences in the ecosystem roles and impacts of different strains/sub-species.

### Co-occurrences of bacterial ASVs and correspondence with habitat

**Fig 4:**
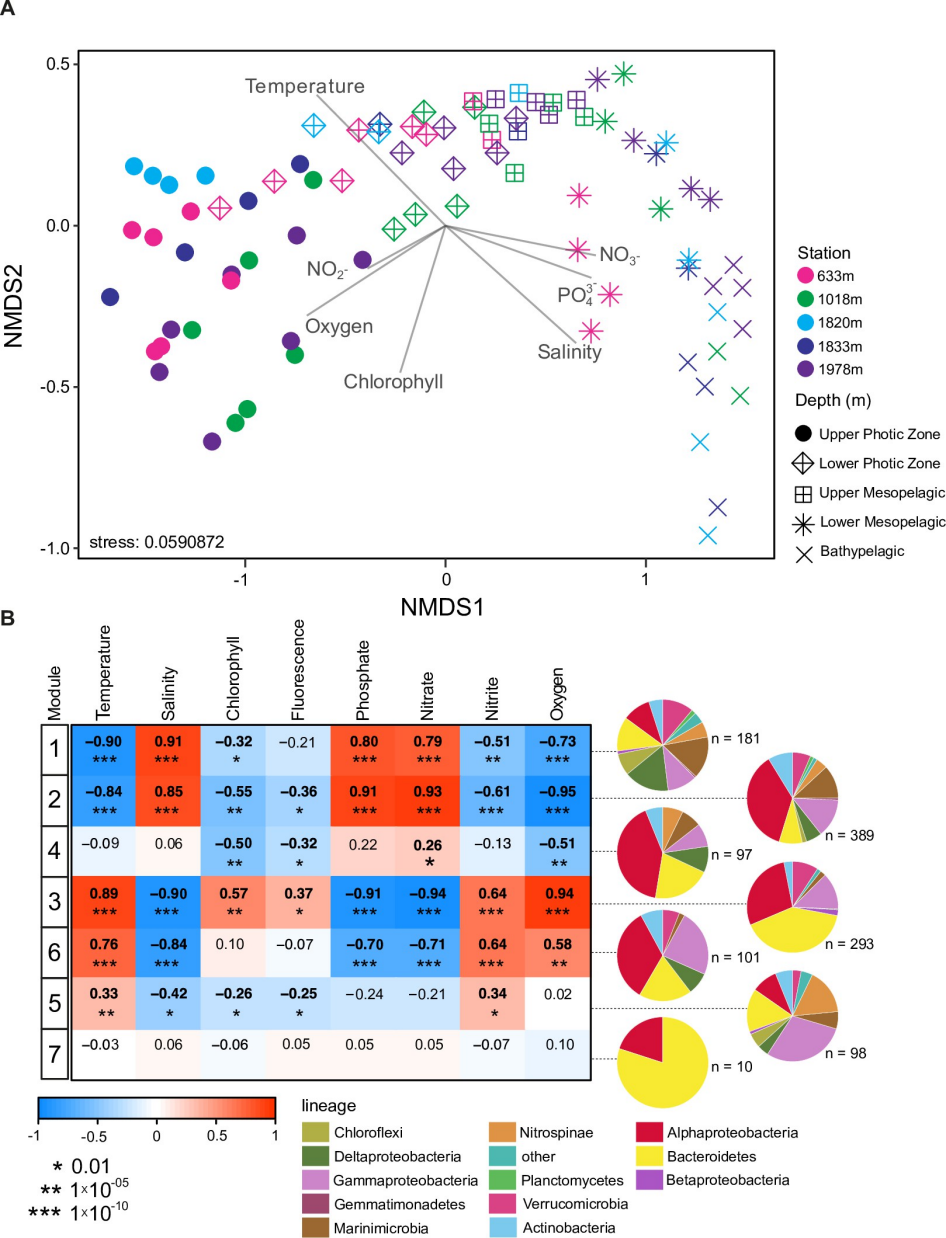
Bacterial community and ASV relationships with habitat. (A) NMDS exploring the relationship between communities within samples from the five refined depth zones (see Fig 3) and environmental parameters. (B) Pearson correlations relating to untransformed environmental physiochemical parameters to the eigengene (first principal component of the abundance matrix) of each WGCNA module. Positive relationships are in red and negative relationships are in blue (as indicated by the color bar). The top number is the Pearson correlation value and asterisks in parenthesis represent the *P*-value for each relationship. Bolded numbers are significant correlations (*P*<0.01; *, <0.01; **, <1x10^-5^; ***, <1x10^-10^). Relationships were examined between the 7 modules and temperature (°C), salinity (PSU), chlorophyll (mg m^-3^), fluorescence, phosphate (μM), nitrate (μM), nitrite (μM), and oxygen (mg L^-1^). Pie charts show the taxonomic groups within each of the 7 modules of co-occurring bacterial ASVs that emerged from WGCNA based on taxonomy assigned using the SILVA database (S4 Table).

Two other types of analyses were performed to examine bacterial communities in the context of their habitat. The first was an NMDS, which showed similar trends as seen using hierarchical clustering, wherein samples from depth zones generally grouped together, and the 900 m samples aligned in the first dimension with other bathypelagic samples (Fig 4A). Additionally, after taxonomic characterization, WGCNA was used to identify highly correlated ASVs across samples from the full dataset and relate the resulting modules (or subnetworks) of co-occurring bacterial taxa to environmental parameters [47, 75]. Seven major modules were identified based on the co-occurrence of ASVs, with ASV numbers ranging from 10 to 389 per module (Fig 4B and S4 Table). Six modules showed varying degrees of correlation with changes in environmental conditions, specifically temperature, salinity, chlorophyll, fluorescence, phosphate, nitrate, nitrite, and oxygen. Silicate was excluded from this analysis as almost all of the samples from May 2016 did not measure silicate, and due to this missing data too many samples would have been excluded by WGCNA. One module (Module 7) did not connect with the environmental data analyzed and consisted of only 10 ASVs, including ASVs classified as Rhodobacteraceae, Flavobacteriaceae, and was the only module to include Cyclobacteriaceae (4 ASVs). For four modules (Modules 1 through 4, see below), the environmental parameter correlations followed depth-related vertical trends in the water column.

Two WGCNA modules and their members exhibited similar statistically significant correlations (*P*<0.05) with respect to the included environmental parameters. Specifically, Module 1 and Module 2 were inversely related to temperature, chlorophyll, fluorescence, nitrite, and oxygen, while positively related to salinity, phosphate, and nitrate (Fig 4B and S1 Table). The negative correlation to chlorophyll and fluorescence was less strong than to the other environmental parameters, and more strongly negative for Module 2 than for Module 1. This indicates that the taxa making up these modules are relatively well-adapted to the deep ocean, which has similar relationships to these parameters, as well as other attributes not quantified herein, such as low availability of labile organic carbon and relatively low productivity. The actual niches of the diverse individual taxa cannot be strictly defined from our limited environmental data, which may covary with other parameters. Nevertheless, the slight difference between Modules 1 and 2 suggests that distinct sets of environmental parameters (or covarying factors) correlate with the different sets of bacterial taxa. These distinctions require further investigation to understand the functional implications.

We then restricted our analysis to the ASVs most highly correlated with the co-occurring ASVs in a module based on Module Membership (MM) > 0.80; where an MM value close to 1 indicates an ASV was highly correlated with its module [47]. Module 1 consisted of 167 highly correlated ASVs and Module 2 consisted of 303 such ASVs, corresponding to 92% and 78% of the total ASVs in these modules, respectively. Module 1’s ASVs were diverse, with 15% of ASVs being classified as Marinimicrobia, 11% SAR324, 10% Pelagibacterales, 12% Bacteroidetes, 11% Verrucomicrobia Arctic97B-4 marine group, 8% Chloroflexi SAR202, and 5% Acidimicrobiales Sva0996 marine group (Fig 4B). Module 2 differed with Pelagibacterales being more dominant than in Module 1, comprising 35% of the ASVs, while also containing 12% Marinimicrobia, 5% SAR324, and 4% Nitrospina ASVs. Between Module 1 and Module 2, there were 31 taxonomic groups that had considerable representation in both modules, but consisting of distinct ASVs (within those groups, S4 Table), including Pelagibacterales, Marinimicrobia, Bacteroidetes, SAR324, Oceanospirillales and Salinisphaerales, and Verrucomicrobia Arctic97B-4 marine group. Thus, overall these two modules are diverse and primarily associated with deep waters. Given that they have similar correlations to environmental comparisons (except chlorophyll and fluorescence), this indicates potential interactions or intra-connectedness between the microbes may be driving the differences in the microbial network.

Module 3 exhibited opposing patterns to those of Modules 1 and 2, as it was significantly negatively related to salinity, phosphate, and nitrate and positively correlated to temperature, chlorophyll, fluorescence, nitrite, and oxygen (Fig 4B). These relationships point to general characteristics of the surface ocean, including presence of phytoplankton and their exudates, as having importance in shaping the taxa in this Module. There were 188 highly correlated ASVs of Module 3 (MM > 0.80; 64% of Module 3 ASVs). The greatest percentage of ASVs in the complete Module 3 set was assigned to Flavobacteriales (39%), including a predominance of Cryomorphaceae (12% of ASVs) and Flavobacteriaceae (21% of ASVs; S4 Table). Rhodobacteraceae were also abundant, making up 17% of the Module 3 ASVs. Pelagibacterales made up 6% of Module 3 ASVs, while there were no ASVs classified as SAR324.

Module 6 was significantly positively correlated to temperature, nitrite, and oxygen and inversely correlated with salinity, phosphate, and nitrate—a similar correlation pattern to Module 3 (Fig 4), but, in contrast, did not have a significant relationship with chlorophyll. These results suggest this module comprises taxa preferring the conditions of surface seawater in which there are variable amounts of phytoplankton (and/or or potential types), such as oligotrophic periods like summer or winter. There were 86 highly correlated ASVs in Module 6 (85%), 24% classified as Oceanospirillales, 16% as Pelagibacterales, 9% as SAR324, 9% as Rhodobacterales, and 7% as Rhizobiales and Rhodospirillales. At the phylum level there were clear differences from Module 3, and even within phyla differences could be observed, such as Oceanospirillales formed only 5% of ASVs in Module 3 versus 24% in Module 6 (S4 Table).

The correlations of Module 4 were much weaker and significant only with chlorophyll (and fluorescence) and oxygen, both negatively, as well as nitrate (positively; Fig 4B), and not correlated to temperature and salinity. This suggests that the taxa comprising Module 4 are capable of thriving across a range of temperatures and salinities, and adapted to conditions characterized by moderately low oxygen, and nitrate. This pattern aligns with the environmental conditions typically found in mid-water, where mixing of different water masses with highly variable temperature and salinity occurs. There were 90 highly correlated ASVs (93%), 41% classified as Pelagibacterales, 16% as Flavobacteriales, 10% as SAR324, and 8% as Nitrospinaceae.

Module 5 showed significant but weak positive correlations with temperature and nitrite, and negative correlations with salinity and chlorophyll (and fluorescence; Fig 4). No correlation was observed with oxygen, nitrate, and phosphate, suggesting that the taxa in this module are associated with conditions in low-productivity mid waters. The most highly correlated ASVs of Module 5 (79 ASVs, 81%) were diverse and included 20% ASVs classified as Nitrospinaceae, 15% as Salinisphaeraceae, 10% as Oceanospirillales, 5% as SAR324, and 3% as Pelagibacterales.

Overall, the co-occurrence patterns herein suggest the presence of a limited number of distinct microbial community ‘states’ in relation to a set of relevant environmental parameters. The functional characteristics of these communities may vary but should ultimately be possible to identify. Clearly, the power to define the niches based on the environmental parameters quantified herein is limited, and numerous other attributes may co-occur that could help distinguish the modules recovered more clearly. These include the amount and composition of organic carbon, and a large number of other elements or compounds (such as vitamins [9]). The modular and co-occurrence patterns observed here must be interpreted with the understanding that within each of these modules there may be numerous other states, overwhelmed by sharp gradients of the parameters from the entire dataset. Within these potential states, beyond the scope of our study, there may be other statistically significant microbial co-occurrence patterns that can be distinguished, which may represent microbial interactions. Our analysis cannot distinguish biological microbe-to-microbe co-occurrence patterns (ASV-to-ASV) from those that are ‘simply’ co-occurring in connection to environmental conditions (ASV-to-environmental parameter). Recently, several studies have revealed that microbe-to-microbe co-occurrence patterns in the ocean, potentially representing biological interactions, can be ‘disentangled’ from those that reflect co-occurrence due to correlations to environmental parameters [76, 77]. Understanding how taxa within the different modules interact competitively or synergistically, and impact ecosystem processes, is a major frontier for study.

### Phylogenetic analysis of Pelagibacterales and SAR324

Thus far our analyses pointed to important roles for Pelagibacterales across the water column, and for SAR324 especially in deeper waters, though also occasionally in the photic zone, with both exhibiting numerous ASVs co-associated with other taxa. Most analyses have relied on SILVA based placement for taxonomic assignment, and we now sought to connect our observations to evolutionary aspects of these two diverse bacterial lineages by characterizing our amplicon sequences using phylogenetic methods. To this end, we performed phylogenetic reconstructions on both these groups that incorporated near full-length 16S rRNA gene sequences obtained from aquatic environments from previously unrecognized members, alongside known reference sequences.

**Fig 5:**
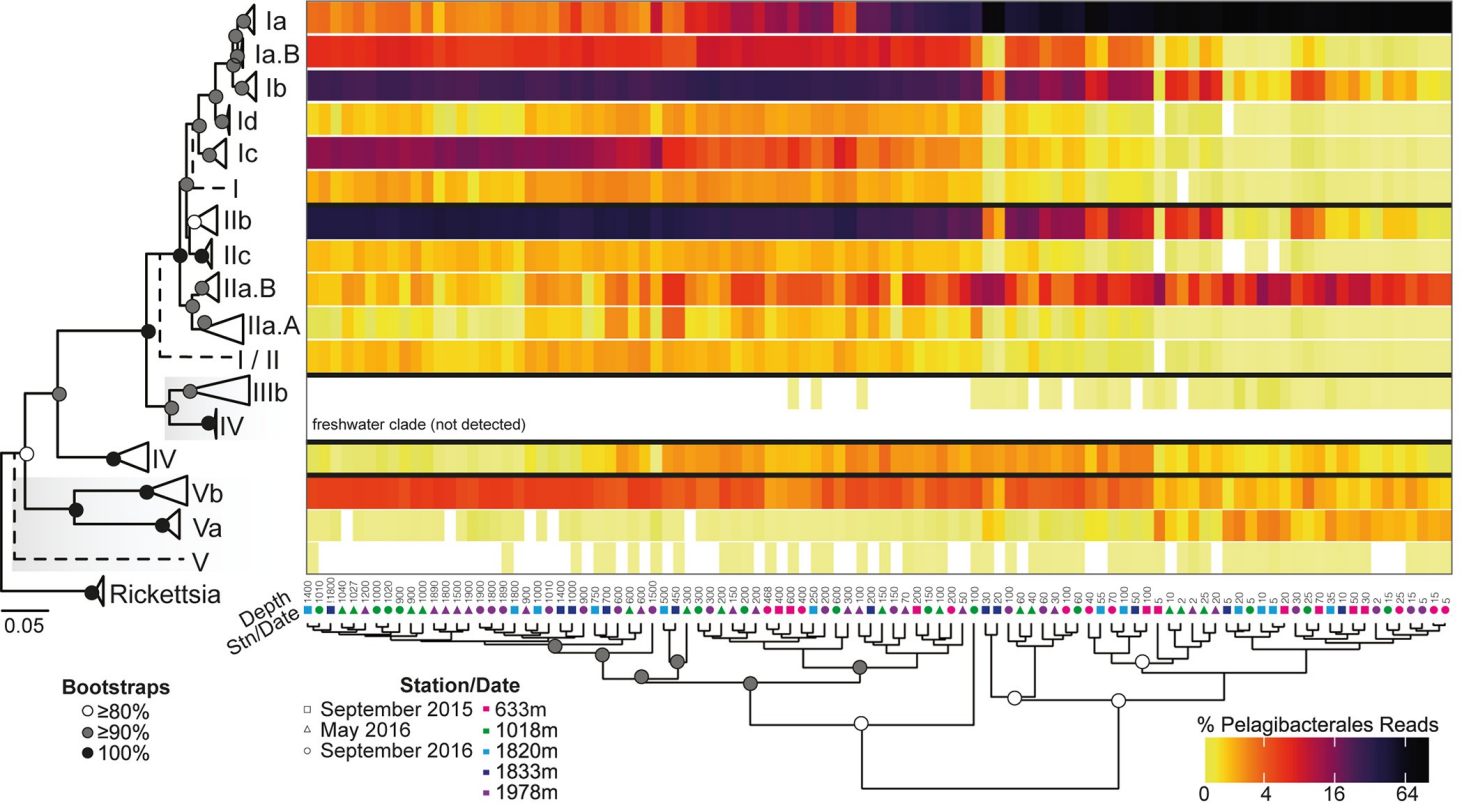
Phylogeny and distributions of Pelagibacterales. The 16S rRNA gene phylogenetic reconstruction utilized full-length sequences and identifies 10 recognized subclades and 3 previously unrecognized subclades, all with bootstrap support >90% (1,000 replicates) except subclade IIb (86% support; see also S4 Fig). Note that dashed lines indicate LCA placements at unsupported nodes, some with relatively few amplicons. For example, that demarked ‘Clade V’—with a dashed line comprised >0.01% of amplicons . Some others with dashed lines were notable, but lacked full length sequences and therefore references sequences were not in the reconstruction. Also shown is the relative abundance of Pelagibacterales subclades as percent of all Pelagibacterales ASVs in each sample for the Monterey Bay Stations and depths sampled. *Pelagibacter* amplicons were first retrieved using PhyloAssigner and a global 16S rRNA gene reference tree [17]. These amplicons were then run in PhyloAssigner using the above Pelagibacterales reference tree. Heat map columns represent individual samples ordered by hierarchical clustering based on the Bray-Curtis similarities of the Pelagibacterales community composition. White in the heat map indicates not detected. Stations (color) and sampling dates (shape) are indicated as is sample depth (m) for each column.

For the Pelagibacterales, the resulting tree topology (Fig 5 and S4 Fig) had similarities to prior phylogenetic analyses [14, 17, 73, 78–80]. However, three supported, previously unrecognized subclades were exposed, and disrupted prior branching structures: Ia.B, Id, and IIc. To ensure that new subclades did not result from possible PCR-related errors, we required that each new subclade contains sequences from at least two different samples. Clade I has three previously identified 16S subclades: Ia and Ib, consistently found in the photic zone [13, 20] and Ic, mostly found in the mesopelagic and bathypelagic [14, 80]. We delineated two additional clade I subclades, named here Ia.B and Id, the former is sister to subclade Ia and the latter branches as sister to a group comprising Ia, Ia.B, and Ib. The third subclade (IIc) recognized herein clusters together with IIb. In our reconstruction, subclade IIa branched as sister to a statistically supported group containing all clade I subclades and subclades IIb and IIc. Compared to clades identified in prior publications, clade IV and clade V (subclades Va and Vb) exhibit significant genomic divergence at both the nucleotide and protein levels [81, 82], and they branched basally to the above groups as initially described [17, 78]. Information on the distributions and apparent niches of the known and newly identified clades are discussed below (*Distributions of phylogenetically resolved Pelagibacterales)*.

**Fig 6:**
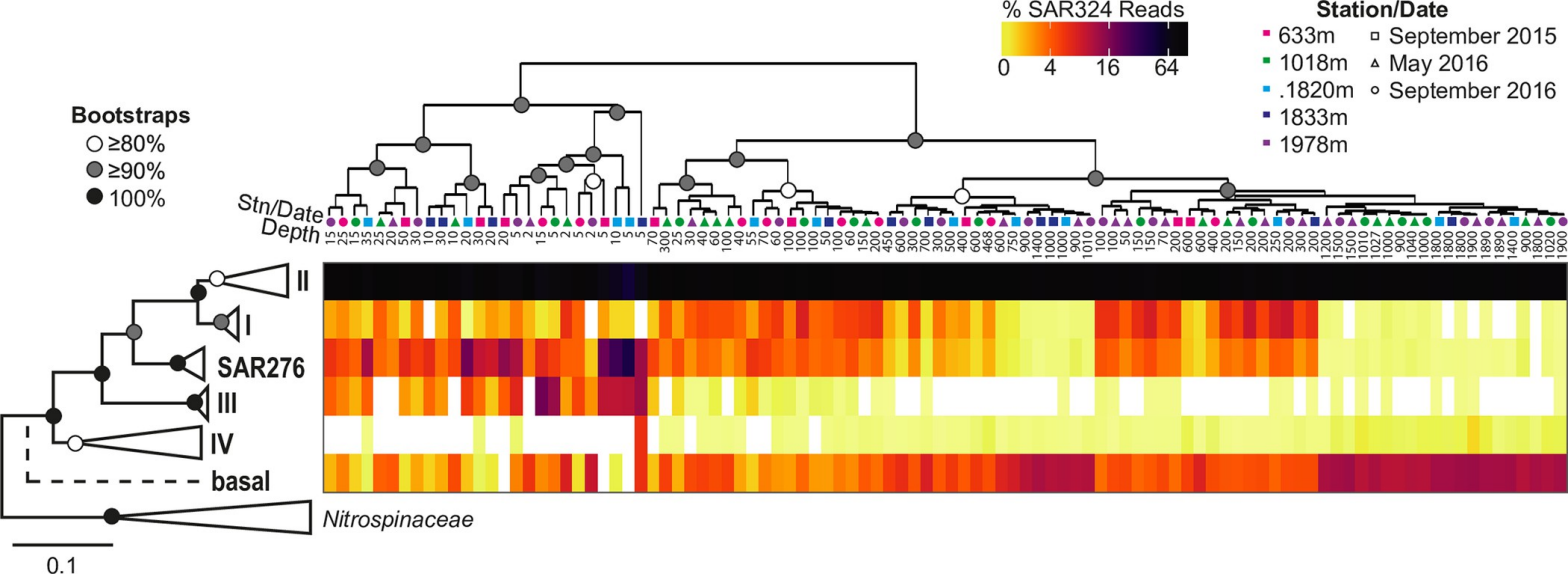
SAR324 16S rRNA gene reference tree identifies novel clades and distributions. Shown is the ML reconstruction with percent relative abundance of SAR324 clade members out of total SAR324 amplicons at the study sites shown to the right of the tree topology. All clades retained support >90% apart from clades II and IV (82 and 88%, respectively; see also S5 Fig). Amplicons were phylogenetically placed on the SAR324 reconstruction developed herein using PhyloAssigner [17] after initial assignment and retrieval using a global 16S rRNA gene reference tree [17]. Heat map columns represent individual samples ordered by hierarchical clustering based on the Bray-Curtis similarities of the SAR324 composition. White in the heat map indicates not detected. Stations (color) and sampling dates (shape) are indicated as is sample depth (m) for each column.

We also performed a 16S rRNA gene phylogenetic reconstruction of SAR324 (Fig 6 and S5 Fig). The resulting tree topology corresponds to previously published topologies [27, 25, 30, 83, 84]. The original three clades of SAR324 based on ITS reconstruction (Brown & Donachie, 2007) were also resolved here with the 16S rRNA gene. We identified two putative basal clades potentially affiliated with this lineage, named here clades III and IV. Our reconstruction places clade III outside the three original SAR324 clades. Clade IV contains sequences included in previous phylogenetic reconstructions, but never as a distinctive or supported clade [30, 83]; distributional data helps to identify the respective niches of these clades (see below).

### Distributions of phylogenetically resolved Pelagibacterales

The Pelagibacterales are known for high diversity [78], something that has been noted in many studies. High microdiversity has been further highlighted at SPOT, where the most temporally common taxa, such as Pelagibacterales, were reported to have the highest microdiversity, quantified therein as the number of 100% ASVs within a given 99% OTU [85]. Here, the 16S rRNA gene Pelagibacterales phylogenetic reconstruction allowed placement of Pelagibacterales amplicons from our study region and examination of community structure as well as subclade relative contributions to the Pelagibacterales collective. Subclade Ia had the highest relative abundance among all subclades and dominated the photic zone Pelagibacterales community (77±13%). This observation is in line with a recent study that found subclade Ia to be the most abundant Pelagibacterales subclade in the surface ocean [18]. Subclade Ib was represented by 27±4% and 24±2% of mesopelagic and bathypelagic Pelagibacterales amplicons, respectively. In the Red Sea subclade 1b is present in both mesopelagic and bathypelagic waters [79] and at the Bermuda Atlantic Time-series Study (BATS), it is usually found in mixed water during late spring and early summer, while Ic is found in deeper water [17, 80]. We observed similar patterns, with subclade Ic exhibiting high relative abundances in the bathypelagic (14±1%), but less in the mesopelagic (4±3%) and the photic zone (<1%). The newly identified subclades exhibited relative abundances that rivaled subclade Ic, with subclade Ia.B having the highest relative abundances among Pelagibacterales in the mesopelagic (10±2%) and bathypelagic (10±1%). Subclade Id exhibited a lower presence than subclade Ia.B, with their highest relative abundance in the mesopelagic (1±0%), with a decrease in relative abundance in the bathypelagic (0.7±0.3%) and photic zone (0.2±0.2%). This suggests that these two new subclades are adapted to the cold, low organic material availability environments seen at deeper depths. Even with statistical support of the Pelagibacterales topology, which reflects those taxa captured thus far by culturing or full-length sequencing, some amplicons were classified as being related to the Last Common Ancestor (LCA) of clade I (indicated with dashed line inserted in phylogenetic tree topology, Fig 5) in the photic (0.2±0.2%), mesopelagic (1±0.3%), and bathypelagic (1±0.3%) zones. Further efforts to characterize these taxa by retrieving their full length 16S rRNA gene sequences would facilitate studies of the affiliation and diversity of these novel ASVs.

Pelagibacterales subclade IIb dominated the mesopelagic (30±8%) and bathypelagic (40±3%) and was less relatively abundant in the photic zone (4±5%), while subclade IIc was detectable at ≤1% relative abundance in all three zones, suggesting this rare type occupies a niche that is conserved across all depths. The similar relative abundances of subclade IIb in the mesopelagic and the bathypelagic connects to the upper mesopelagic depth distributions that have been reported at BATS during spring [13, 86] and also in the deeper cold Arctic Ocean [19]. Subclade IIa relative abundance was an order of magnitude lower (8±3%) than that of subclade Ia in the photic zone and decreased further to 5±1% and 2±1% of Pelagibacterales in the mesopelagic and bathypelagic, respectively. Our analysis of IIa sequences supports the further breakdown into two subclades [87], with subclade IIa.B exhibiting higher relative abundances in the photic zone (5±4%), decreasing in relative abundance in the mesopelagic (2±2%) and bathypelagic (0.4±0.2%). Subclade IIa.A was found at low abundances throughout the water column, with its highest relative abundance in the mesopelagic (0.6±0.4%) and even still being detectable (at <0.2% relative abundance) in the photic zone as well as the bathypelagic. While there are fewer IIa.A sequences in our dataset compared to other Pelagibacterales, their relative abundance was generally higher at depths where oxygen values are lowest within the water column, in agreement with reports of IIa.A higher relative abundances in Oxygen Minimum Zones (OMZs) [87]. A small number of amplicons were classified as being basal to clade I/II in the photic (0.1±0.2%), mesopelagic (1±0.3%), and bathypelagic (1±0.2%) (Fig 5).

While clades III, IV, and V were among the least relatively abundant in our samples, they did align with specific environmental conditions encountered in our sample set–or were not encountered at all, in accordance with prior knowledge of habitats. Subclade IIIa, which is usually abundant in brackish water [88], formed 0.05±0.07% of photic zone Pelagibacterales amplicons and was not detected in the mesopelagic and bathypelagic. Subclade IIIb (LD12) is a known freshwater subclade and was not detected in any samples. In contrast, Clade IV was detected throughout the water column, generally at ≤1% relative abundance, but, compared to its relative contributions in the photic zone and bathypelagic, was more important in the mesopelagic. Clade V showed slightly higher contributions, with Subclade Va showing <0.1% relative abundance in the mesopelagic and bathypelagic, but was an order of magnitude higher in the photic zone (still <1%). Subclade Vb at 3±1% and 4±0% in the mesopelagic and bathypelagic, respectively, and <1% in the photic zone. Adding to the established distribution of subclade Vb in the surface ocean [7], our study found subclade Vb at all depths throughout the water column. Overall, the broad similarity of our phylogenetics-based analysis of SAR11 ASVs and their relative abundances suggests conserved niches for the clades across and between ocean basins, a trait that is ripe to be systematically evaluated using phylogenetic approaches, such as those presented herein.

### Patterns in phylogenetically resolved SAR324 clades

Analysis of our 16S rRNA gene amplicons against the SAR324 reference tree, using PhyloAssigner [17], augments previously described vertical distributions. While considered ubiquitous below the photic zone [24–27], SAR324 has been described in many marine environments such as the bottom of the mixed layer in the Sargasso Sea and northeastern subarctic Pacific Ocean [21], throughout the water column in the North Pacific Subtropical Gyre [25, 29, 84], the Saanich Inlet OMZ [89], and coastal polar waters during winter and deep polar waters year-round [24], as well as in marine hydrothermal systems [30, 83]. A phylogenetic examination of the clade abundances of SAR324 amplicons identified clade II as the dominant clade across all depths (photic: 87±10%; mesopelagic: 91±2%; bathypelagic: 90±1%) (Fig 6), as indicated from the original observations that distinguished Clade II from the North Pacific and other locations [28]. Clade I was found predominantly in the mesopelagic (3±2%), with lower contributions in the photic zone (2±1%) and bathypelagic (<0.1%). Low Clade I contributions in the photic zone are consistent with results from BATS [90]. Clade SAR276 decreased in relative abundance with depth (Fig 6), making up 6±8% of SAR324 in the photic, 2±1% in the mesopelagic, and <0.1% in the bathypelagic; a preference for photic zone depths is consistent with previous results from the North Pacific and other marine regions [25], while SAR276 was relatively stable (2–3%) across the photic and mesopelagic at BATS where it was the only clade with significant abundance in the photic zone. Clades III and IV occurred at very low abundances throughout the water column. Clade III was mostly found in the photic zone (3±4%) with low detection (<0.01%) in the mesopelagic and bathypelagic. Clade III has a potentially photic zone distribution, consisting of sequences from the photic zone as well as being detected in our photic zone samples, though was also detected in a few samples from the mesopelagic and bathypelagic. Clade IV consists of sequences from sediment and subsurface environments [91–94]. In our dataset, clade IV was found in the photic (0.1±0.7%) and bathypelagic (0.2±0.1%) with minimal detection in the mesopelagic (0.07±0.06%). Our observation that there are distinct differences in depth distributions of SAR324 groups is similar to observations of depth differences observed based on average nucleotide identity of partial SAR324 genomes (classified as groups ’A’ to ‘E’) from the North Pacific Subtropical Gyre [29], which were related to functional differences. Similar to the SAR11 analyses above, this indicates remarkable niche similarity within and between ocean basins at least as experience by members of the various SAR324 clades. While the newly identified subclades III and IV are not yet connected to functional information, their distributional data should help targeting in future studies, given that Clade III is found primarily in the Monterey Bay photic zone and Clade IV is seen throughout the water column. Additionally, it will be important to resolve (as possible) the 16S rRNA phylogeny in connection to SAR324 phylogenomics and genome taxonomy. Finally, even with statistical support of the SAR324 topology, a notable number of amplicons were classified as being related to the Last Common Ancestor of SAR324 (“basal”; based on reference data availability), especially in the mesopelagic (7±1%) and bathypelagic (10±1%). Full length 16S rRNA gene sequences, or genome sequences, would help to resolve their affiliation and diversity. Here, hierarchical clustering shifted from the general depth-related consistency seen between the overall community composition (Fig 3) and the SAR11 group (Fig 5), indicating that greater partitioning of clade II likely exists but has not been phylogenetically resolved yet. Additional full-length sequences or increased genomic data from phylogenomic analyses will be important for resolving whether these are most closely related to the SAR324 Last Common Ancestor or belong to existing clades.

## Conclusions

Our in-depth descriptions, identification of significant statistical WGCNA-based modules, and evolutionary analyses revealed patterns in vertical distributions of bacterial groups throughout the water column in Monterey Bay. Together with decades of biological oceanographical measurements [52], the data herein provide a baseline from which to study long-term changes to the overall health, nutrient cycling, and ecological change in the Monterey Bay. The microbial community composition is regulated by a combination of the co-occurrence of other microbes and the physical and chemical environment. Our phylogenetic reconstructions demonstrate evolutionary diversity that is not fully distinguished by the V1-V2 ASV analysis within the highly abundant, well-defined Pelagibacterales, as well as SAR324 bacteria, for which the ecological ramifications await discovery. Overall, these results emphasize the importance of identifying evolutionary diversity and how it might connect to ecological differences. The results further stress that the organisms represented as being most abundant by amplicon analyses are largely unrepresented in culture collections, and hence have still unknown ecologies. Our study, alongside ongoing and future efforts, provides a path for understanding the niches of these organisms, and eventually implications of their distinctive distributions and how they relate to ecosystem function.

## Supporting information

S1 Table Sampling information and environmental parameters of seawater samples from the surface ocean to the seafloor. Also included are cell counts from flow cytometric analysis.
(XLSX)

S2 Table V1-V2 16S rRNA gene amplicon sequencing information for each sample: Number of raw reads (paired-end), number of amplicons (post quality control), number of amplicon sequence variants (ASVs, with cyanos removed). Illumina plate indicates which samples were sequenced simultaneously.
(XLSX)

S3 Table Amplicon counts and taxonomy for the top 50 most relatively abundant V1-V2 16S rRNA gene ASVs per sample and the corresponding ASV sequence. (XLSX)

S4 Table Module composition and taxonomic classification of members ASVs. (XLSX)

S1 Fig Rarefaction curves of sequencing depth versus number of observed bacterial ASVs per sample. Rarefaction curve final slope vs. (A) depth and (B) total number of amplicons. Colored shapes indicate the station (color) and sampling date (shape) of each sample. (C) Rarefaction curve of number of ASVs vs. amplicons. Each station (color) plotted separately.
(PDF)

S2 Fig Shannon diversity of samples. (A) Shannon diversity computed using non-rarefied data (Fig 2C examines rarefied data). (B) Shannon diversity versus the number of amplicons per sample indicates there is not a correlation between these two factors (a regression, which is not mathematically appropriate to this data, renders R^2^ value of 0.059. It is also important to remember that here and throughout sequencing depth differences should always be kept in mind.
(PDF)

S3 Fig Hierarchical clustering of samples based on bacterial ASV Bray-Curtis dissimilarities from Fig 3 with sample names indicated. Multiscale bootstrap resampling via “pvclust” identified p-values (red) and bootstrap probability values (green) for each cluster.
(PDF)

S4 Fig Taxonomic patterns of highly abundant ASVs in bacterial communities along the depth gradient. (A) Heat map of bacterial ASV relative abundances with cyanobacterial ASVs excluded (149 in total). (B) Hierarchical clustering based on the top 1,000 most relatively abundant ASVs in samples with cyanobacterial ASVs excluded.
(PDF)

S5 Fig Pelagibacterales 16S rRNA gene Maximum Likelihood phylogenetic reconstruction with accession numbers. A total of 137 near full-length 16S rRNA gene sequences representing Pelagibacterales were retrieved from the SILVA database (release 128) and through blastn (v2.6.0) [48] searches of GenBank using sequences from previously published studies. The 137 sequences plus one *Escherichia coli* and one *Magnetococcus marinus* (as an outgroup) sequence were aligned using MAFFT (v7.402) [49] with default parameters and ambiguously aligned sites were removed using trimAl (v1.4) with a no gap threshold [50]. Phylogenetic inferences were performed using Maximum Likelihood methods implemented in RAxML (v8.2.10) [51] under gamma-corrected GTR model of evolution with 1,000 bootstrap replicates based on 1,303 homologous positions.
(PDF)

S6 Fig SAR324 16S rRNA gene Maximum Likelihood phylogenetic reconstruction with accession numbers. Eighty-eight near full-length 16S rRNA gene sequences were retrieved by GenBank blastn (v2.6.0) [48] searches using sequences from previously published trees. These 88 sequences alongside three *Nitrospinaceae*, two *Desulfobulbaceae*, and three *Desulfovibrionaceae* (as an outgroup) were aligned with MAFFT (v7.402) [49] using the L-INS-I algorithm and ambiguously aligned sites were removed using trimAl (v1.4) with a gap threshold of 0.3 [50]. Phylogenetic inferences were performed using Maximum Likelihood methods implemented in RAxML (v8.2.10) [51] under gamma corrected GTR model of evolution with 1,000 bootstrap replicates based on 1,227 homologous positions.
(PDF)
