# Supplementary figures and images for: Gradients of bacteria in the oceanic water column reveal finely-resolved vertical distributions
Source: PLoS One. 2024 Apr 2;19(4):e0298139. doi: 10.1371/journal.pone.0298139 (PMC10986988; doi:10.1371/journal.pone.0298139)

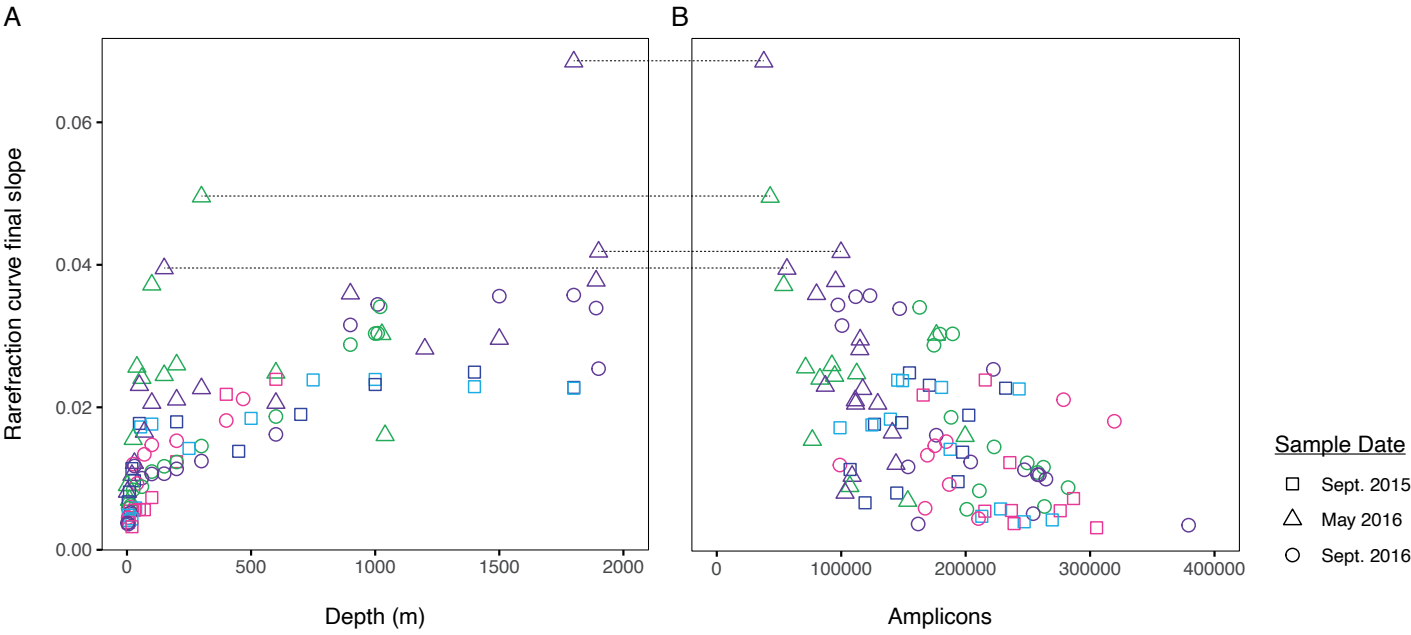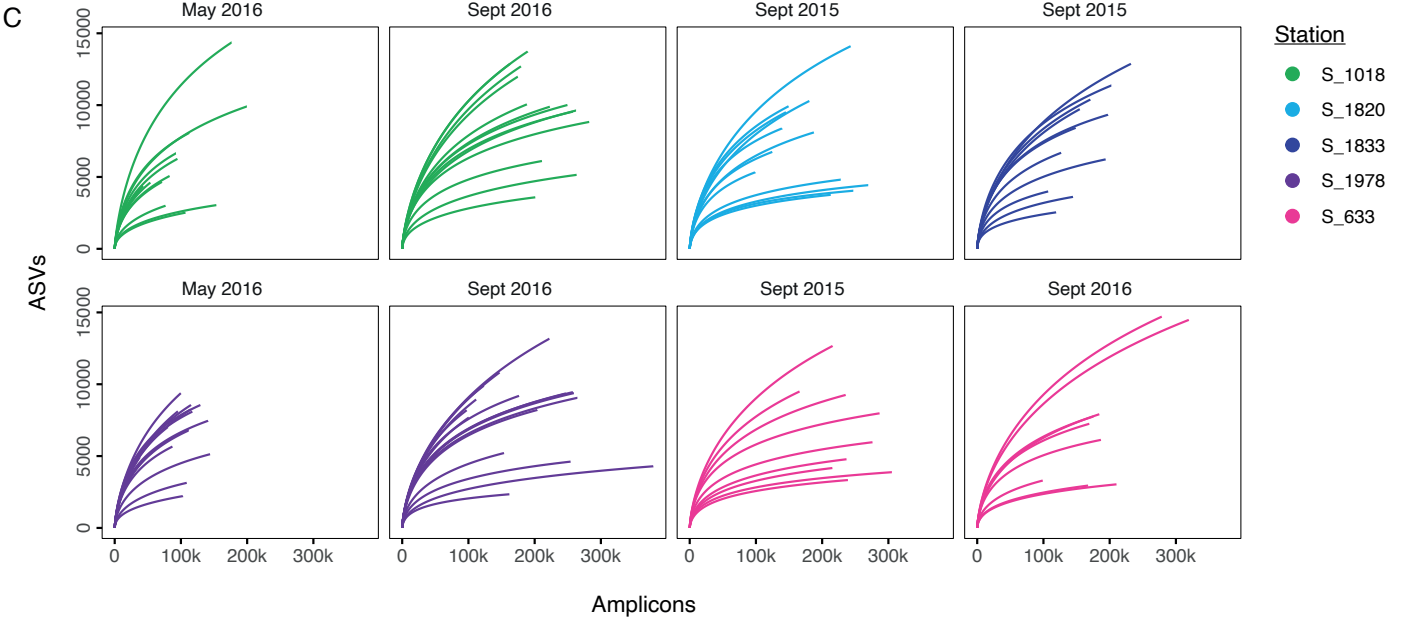

Supplement: S1 Fig — Rarefaction curve final slope vs. (A) depth and (B) total number of amplicons. Colored shapes indicate the station (color) and sampling date (shape) of each sample. (C) Rarefaction curve of number of ASVs vs. amplicons. Each station (color) plotted separately. (PDF) [file pone.0298139.s005.pdf]

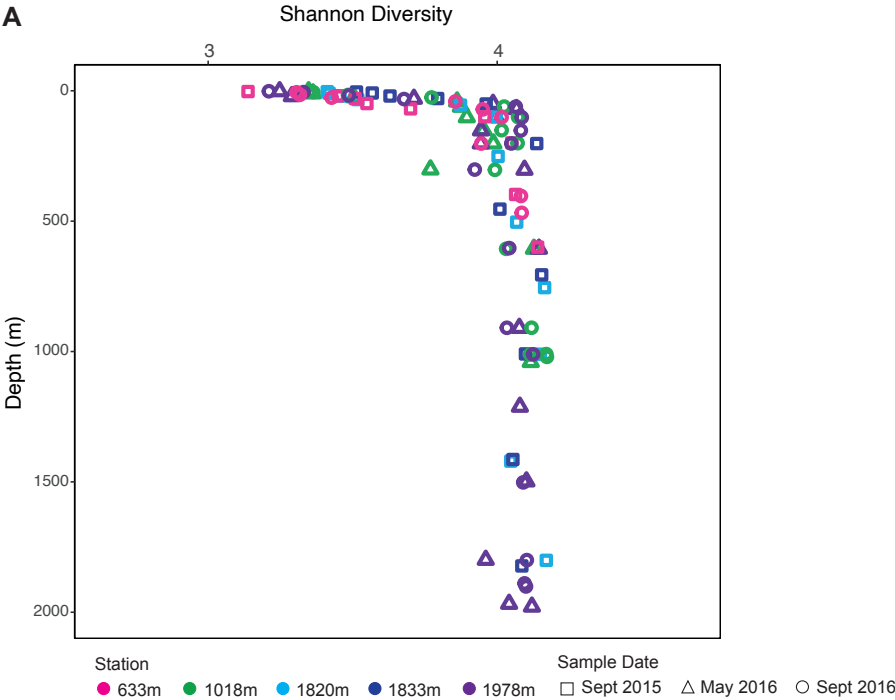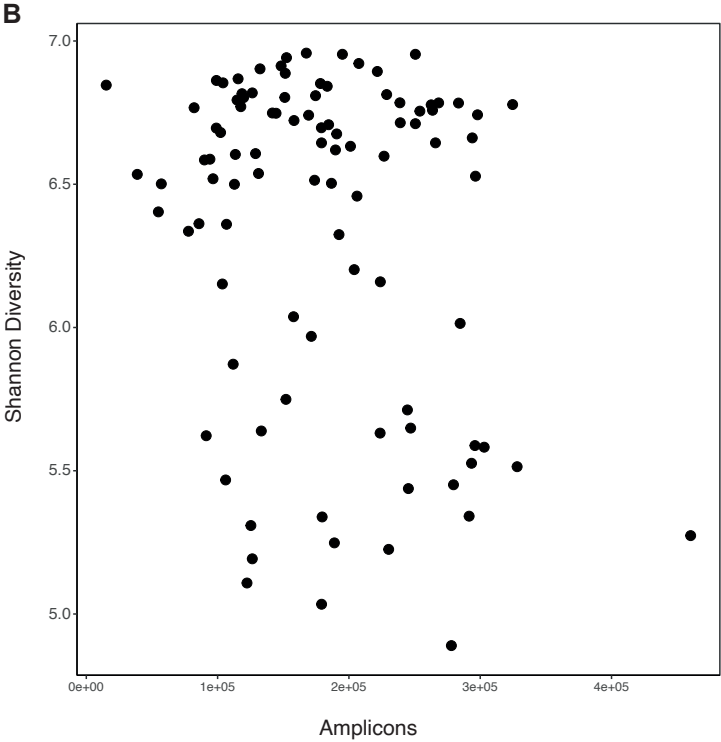

Supplement: S2 Fig — (A) Shannon diversity computed using non-rarefied data (Fig 2C examines rarefied data). (B) Shannon diversity versus the number of amplicons per sample indicates there is not a correlation between these two factors (a regression, which is not mathematically appropriate to this data, renders R2 value of 0.059. It is also important to remember that here and throughout sequencing depth differences should always be kept in mind. (PDF) [file pone.0298139.s006.pdf]

Distance: bray-curtis  
Cluster method: average

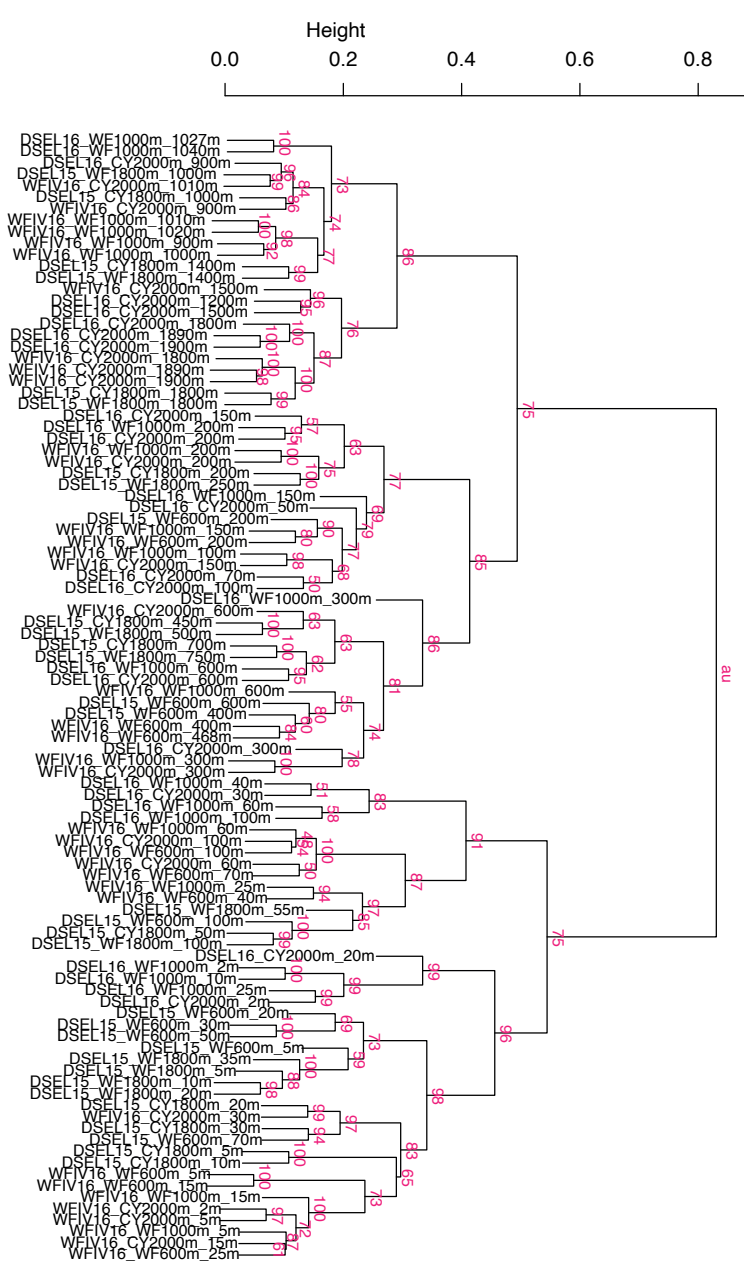

Supplement: S3 Fig — Multiscale bootstrap resampling via “pvclust” identified p-values (red) and bootstrap probability values (green) for each cluster. (PDF) [file pone.0298139.s007.pdf]

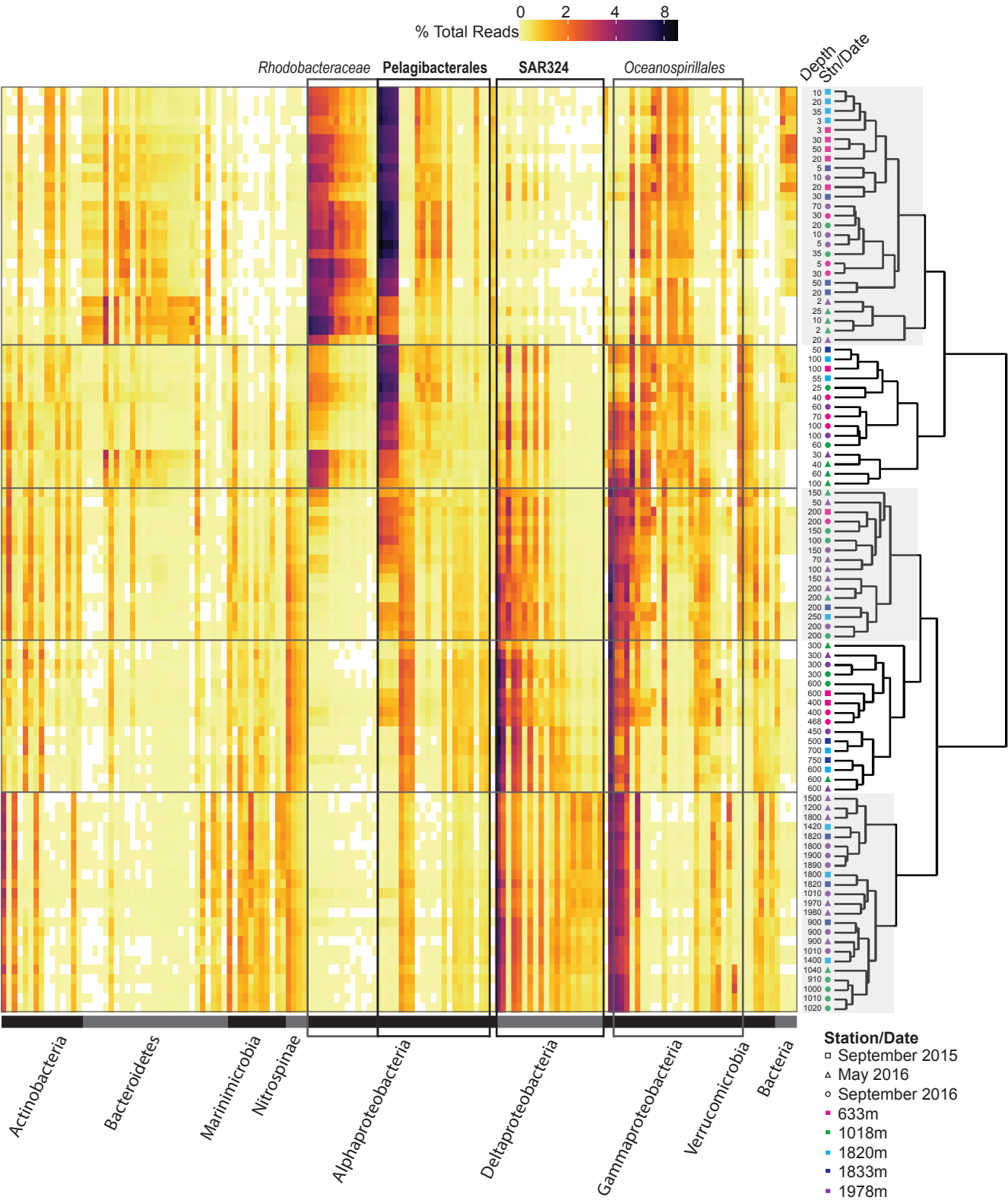

Supplement: S4 Fig — (A) Heat map of bacterial ASV relative abundances with cyanobacterial ASVs excluded (149 in total). (B) Hierarchical clustering based on the top 1,000 most relatively abundant ASVs in samples with cyanobacterial ASVs excluded. (PDF) [file pone.0298139.s008.pdf]

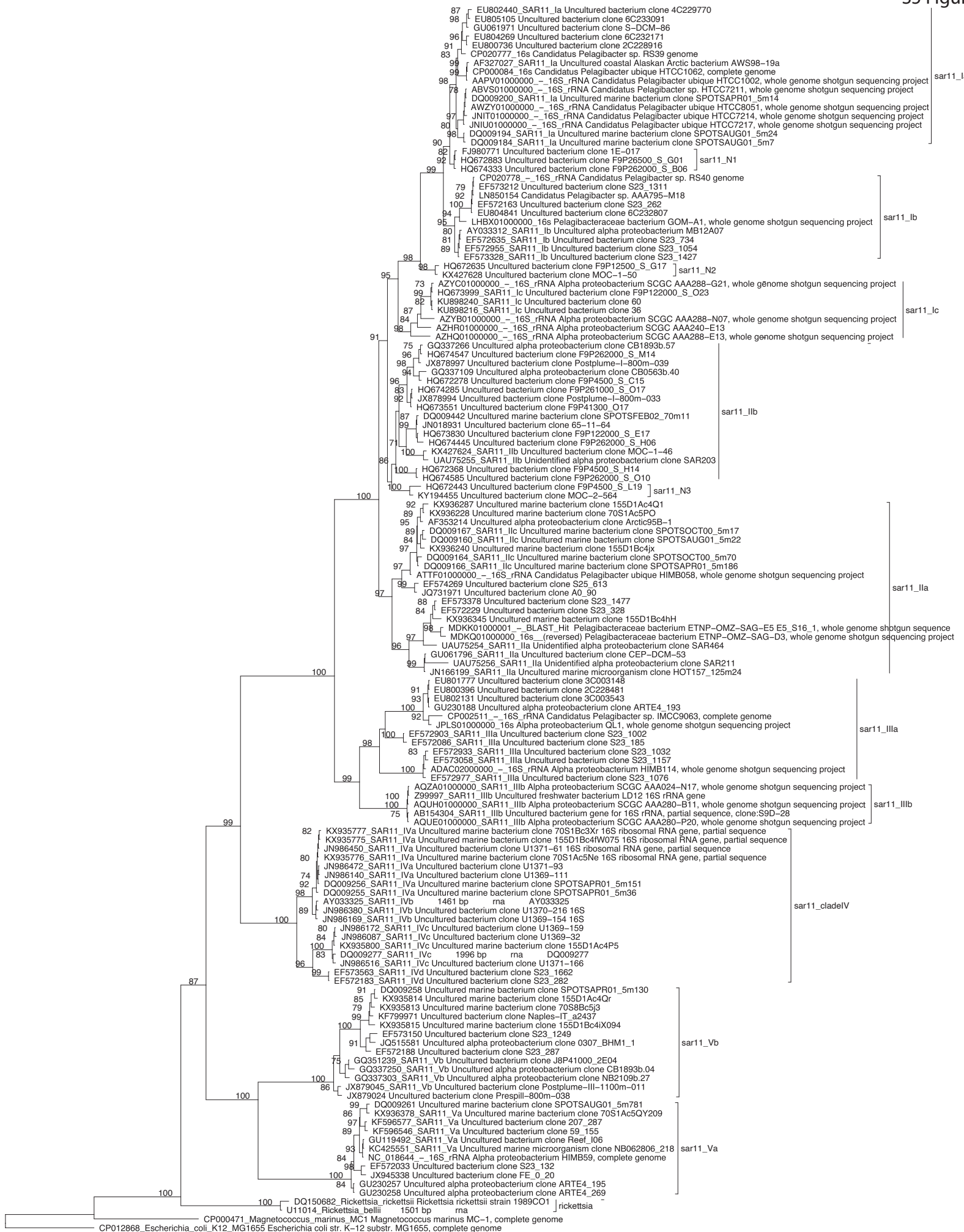

Supplement: S5 Fig — A total of 137 near full-length 16S rRNA gene sequences representing Pelagibacterales were retrieved from the SILVA database (release 128) and through blastn (v2.6.0) [48] searches of GenBank using sequences from previously published studies. The 137 sequences plus one Escherichia coli and one Magnetococcus marinus (as an outgroup) sequence were aligned using MAFFT (v7.402) [49] with default parameters and ambiguously aligned sites were removed using trimAl (v1.4) with a no gap threshold [50]. Phylogenetic inferences were performed using Maximum Likelihood methods implemented in RAxML (v8.2.10) [51] under gamma-corrected GTR model of evolution with 1,000 bootstrap replicates based on 1,303 homologous positions. (PDF) [file pone.0298139.s009.pdf]

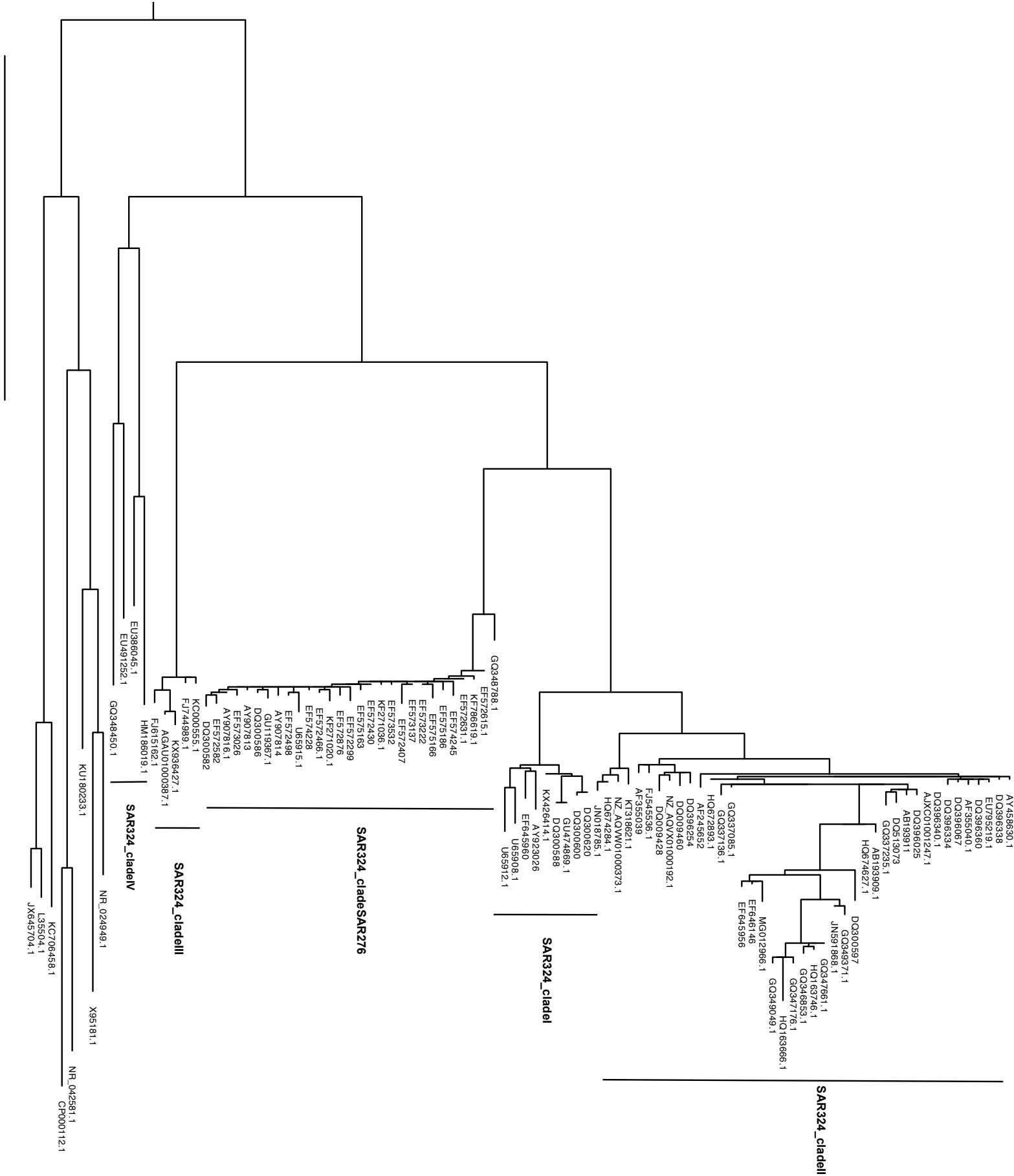

Supplement: S6 Fig — Eighty-eight near full-length 16S rRNA gene sequences were retrieved by GenBank blastn (v2.6.0) [48] searches using sequences from previously published trees. These 88 sequences alongside three Nitrospinaceae, two Desulfobulbaceae, and three Desulfovibrionaceae (as an outgroup) were aligned with MAFFT (v7.402) [49] using the L-INS-I algorithm and ambiguously aligned sites were removed using trimAl (v1.4) with a gap threshold of 0.3 [50]. Phylogenetic inferences were performed using Maximum Likelihood methods implemented in RAxML (v8.2.10) [51] under gamma corrected GTR model of evolution with 1,000 bootstrap replicates based on 1,227 homologous positions. (PDF) [file pone.0298139.s010.pdf]
